# BigFiRSt: A Software Program Using Big Data Technique for Mining Simple Sequence Repeats From Large-Scale Sequencing Data

**DOI:** 10.3389/fdata.2021.727216

**Published:** 2022-01-18

**Authors:** Jinxiang Chen, Fuyi Li, Miao Wang, Junlong Li, Tatiana T. Marquez-Lago, André Leier, Jerico Revote, Shuqin Li, Quanzhong Liu, Jiangning Song

**Affiliations:** ^1^Department of Software Engineering, College of Information Engineering, Northwest A&F University, Yangling, China; ^2^Department of Biochemistry and Molecular Biology, Biomedicine Discovery Institute, Monash University, Melbourne, VIC, Australia; ^3^Monash Centre for Data Science, Monash University, Melbourne, VIC, Australia; ^4^Department of Microbiology and Immunity, The Peter Doherty Institute for Infection and Immunity, The University of Melbourne, Melbourne, VIC, Australia; ^5^Department of Genetics, School of Medicine, University of Alabama at Birmingham, Birmingham, AL, United States; ^6^Department of Cell, Developmental and Integrative Biology, School of Medicine, University of Alabama at Birmingham, Birmingham, AL, United States

**Keywords:** next-generation sequencing, read pairs, Simple Sequence Repeats (SSR), Hadoop, big data

## Abstract

**Background:**

Simple Sequence Repeats (SSRs) are short tandem repeats of nucleotide sequences. It has been shown that SSRs are associated with human diseases and are of medical relevance. Accordingly, a variety of computational methods have been proposed to mine SSRs from genomes. Conventional methods rely on a high-quality complete genome to identify SSRs. However, the sequenced genome often misses several highly repetitive regions. Moreover, many non-model species have no entire genomes. With the recent advances of next-generation sequencing (NGS) techniques, large-scale sequence reads for any species can be rapidly generated using NGS. In this context, a number of methods have been proposed to identify thousands of SSR loci within large amounts of reads for non-model species. While the most commonly used NGS platforms (e.g., Illumina platform) on the market generally provide short paired-end reads, merging overlapping paired-end reads has become a common way prior to the identification of SSR loci. This has posed a big data analysis challenge for traditional stand-alone tools to merge short read pairs and identify SSRs from large-scale data.

**Results:**

In this study, we present a new Hadoop-based software program, termed BigFiRSt, to address this problem using cutting-edge big data technology. BigFiRSt consists of two major modules, BigFLASH and BigPERF, implemented based on two state-of-the-art stand-alone tools, FLASH and PERF, respectively. BigFLASH and BigPERF address the problem of merging short read pairs and mining SSRs in the big data manner, respectively. Comprehensive benchmarking experiments show that BigFiRSt can dramatically reduce the execution times of fast read pairs merging and SSRs mining from very large-scale DNA sequence data.

**Conclusions:**

The excellent performance of BigFiRSt mainly resorts to the Big Data Hadoop technology to merge read pairs and mine SSRs in parallel and distributed computing on clusters. We anticipate BigFiRSt will be a valuable tool in the coming biological Big Data era.

## Introduction

Simple Sequence Repeats (SSRs), also known as short tandem repeats (STRs) or microsatellites (Fan and Chu, 2007; Madesis et al., 2013), are highly mutable nucleotide sequences (Vargas Jentzsch et al., 2013). Previous studies have shown that copy number alterations in tandem repeat DNA are associated with at least 31 different human diseases (Mitsuhashi et al., 2019). As a particular type of tandem repeats, SSRs are also related to many diseases such as colon cancer (Velasco et al., 2019) and humans' neurodegenerative disease (Cao et al., 2014), human triplet-repeat expansion diseases (Caskey et al., 1992; Mitas, 1997). Furthermore, as one of the most popular molecular markers (Guang et al., 2019), SSRs have been widely applied in numerous scientific researches including ecological investigation (Selkoe and Toonen, 2010), human population (Willems et al., 2014), genome evolution (Cavagnaro et al., 2010), plant genetics (Zalapa et al., 2012) and forensic analysis (de Knijff, 2018), and have several biomedical applications (Girgis and Sheetlin, 2013). Notably, repeats in the genome are species-specific (Girgis, 2015), SSRs are likely to be unknown for new genomes. Therefore, SSRs identification in new genomes is fundamentally important for understanding microsatellite evolution mechanisms (Ellegren, 2004).

Conventional experimental methods for SSR identification, such as labeled probes, are often labor-intensive and -expensive (Fernandez-Silva and Toonen, 2013). Computational SSR identification methods provide a valuable and alternative strategy for large-scale experimental design efficiently. Given the importance and value of computational methods for SSR identification, there has been encouraging progress in the development of computational methods and tools for SSR identification. Lim et al. (2013) provided a review of these methods developed before 2013. Various methods/tools have been developed in recent years. These tools are broadly classified into four categories: (i) graphical interface-based methods including GMATo (Wang et al., 2013) and GMATA (Wang and Wang, 2016), (ii) web interface-based methods including ProGeRF (Lopes et al., 2015), QDD (Meglécz et al., 2014), MISA-web (Beier et al., 2017), (iii) database-based methods including SSRome (Mokhtar and Atia, 2018) and MSDB (Avvaru et al., 2017a), and (iv) stand-alone-based methods including SA-SSR (Pickett et al., 2016), Kmer-SSR (Pickett et al., 2017), PERF (Avvaru et al., 2017b), Dot2dot (Genovese et al., 2019) and Look4TRs (Velasco et al., 2019). Most existing methods are generally designed to identify SSRs for species with the entire genome sequence available. These methods rely heavily on a high-quality assembled genome (Guo et al., 2018). However, many non-model species have no entire genomes. Fortunately, new NGS technologies can produce large numbers of genomics data for any species, and this has made it possible to identify SSRs from the newly assembled genome (Andersen and Mills, 2014). However, it is the biggest challenge to assemble a genome using short reads (Magoc and Salzberg, 2011). Moreover, it presents a significant obstacle to aligning reads within the repeat regions to the reference genome (Nashta-ali et al., 2017). As a result, the assembled genome often misses highly repetitive regions (Chu et al., 2016); even good-quality human reference genomes often contain missing bases in repeat regions (Chu et al., 2016). Thus, it is difficult to assemble a high-quality genome (Gnerre et al., 2011; Pickett et al., 2016). In scenarios where the target patterns are very sparse in the genomes, such as clustered repeats like CRISPR region, it is basically wasteful to find the repetitive sequences by assembling all sequencing reads into the genomes (Chen et al., 2019). To address this, in recent years various methods (Castoe et al., 2012; Gymrek et al., 2012; Miller et al., 2013; Fungtammasan et al., 2015; Tang and Nzabarushimana, 2017) have been proposed to identify SSRs from raw sequence data generated by NGS. After identifying SSRs in reads, the non-repetitive flanking sequence of SSR-containing reads can be used to map to the reference for increasing the alignment specificity (Gymrek et al., 2012). Furthermore, analyses of SSRs based on NGS have been used in a range of applications, including forensic analysis (Van Neste et al., 2014; Børsting and Morling, 2015; Parson et al., 2016; van der Gaag et al., 2016; Hoogenboom et al., 2017; de Knijff, 2018; Ganschow et al., 2018), SSRs genotyping (Bornman et al., 2012; Kistler et al., 2017; Budiš et al., 2018), stutter analysis (Vilsen et al., 2018), population genetic (Wirtz et al., 2016) and SSR Markers in Plants (Taheri et al., 2018).

Generally, a typical SSR locus is represented in the repeat modules surrounded by both flanking regions (Budiš et al., 2018). An example of SSR allele is “ACGATGATCGATAGATAGATAGATAGATAGATAGATAGATAGTCAGAGCACC”, which means that the sequences “ACGATGATC” and “GTCAGAGCACC” represent the upstream and downstream region around the motif GATA with eight repeats, respectively. Certain NGS technologies such as Roche 454 could provide reads that fully contain SSRs along with suitable flanking sequences (Perry and Rowe, 2011). In recent years, emerging NGS technologies such as PacBio and Nanopore can produce long reads (Mardis, 2017). However, the most commonly used NGS platforms (e.g., Illumina) on the market often provide short paired-end reads (Escalona et al., 2016; Wang, 2016). In most cases, short reads do not contain full SSR allele regions (Budiš et al., 2018). Thus, constructing longer reads by merging paired-end reads has been used as a common strategy prior to identifying SSR motifs (van der Gaag et al., 2016; Hoogenboom et al., 2017; Ganschow et al., 2018). Several paired-end read merging algorithms have been proposed in recent years, which include FLASH (Magoc and Salzberg, 2011), leeHom (Renaud et al., 2014), PEAR (Zhang et al., 2013), BBMerge (Bushnell et al., 2017) and Konnector (Vandervalk et al., 2014), OverlapPER (Oliveira et al., 2018), Cope (Liu et al., 2012), and XORRO (Dickson and Gloor, 2013). There also exist approaches and tools such as SSRs-pipeline (Miller et al., 2013) and RAD-seq-Assembly-Microsatellite (Xue et al., 2017), which integrate paired-end reads merging and SSRs mining into a single pipeline.

These computational methods and tools have been used for merging paired-end reads and identifying novel SSRs. Other analysis tools such as *iLearn* (Chen et al., 2020, 2021), BioSeq-Analysis (Liu, 2019; Liu et al., 2019) and BioSeq-BLM (Li et al., 2021) were recently developed to handle with the avalanche of biological sequences. However, with the continued development of NGS technologies, there is a strong need to develop new paired-end read merging and SSRs mining methods that better meet the “Big Data” analysis (Wordsworth et al., 2018). As NGS technology can often generate hundreds of gigabytes (GB) sequence data in compressed FASTQ format in every single run (Wang, 2016), it is becoming more and more difficult and time-consuming to use these stand-alone methods and tools to merge paired-end reads and identify SSR loci from such large-scale datasets. To the best of our knowledge, there are currently no methods and tools to date that are developed based on Big Data techniques for merging paired-end reads and mining SSRs. Thus, it would be highly desirable and valuable to significantly enhance the performance of paired-end reads merging and SSRs mining tools by combining the cutting-edge Big Data techniques. In this way, the computational SSRs mining approaches could keep up with the pace of data explosion and efficiently deal with the growth of such large-scale data.

Traditional parallel computing technique based on message passing interface (Gropp et al., 1999) is more effective for moderately sized data and computational-intensive problem (Kang et al., 2015). It is not the best choice to deal with the vast amount of data (Samadi et al., 2018). Hadoop (White, 2009) and Spark (Zaharia et al., 2010) have become two standard big data technologies to handle huge the size of data (Samadi et al., 2018). They have been widely used in the bioinformatics area to deal with the rapid growth and accumulation of biomedical Big Data. Table 1 summarizes the bioinformatics tools developed based on Big Data technologies for handling large-scale sequence data. These methods are involved in many different tasks, including alignment and mapping, sequence analysis, genome analysis, sequence assembly, error correction, duplicate DNA reads and clustering analysis. However, there is no bioinformatics method based on Big Data techniques for merging paired-end reads and mining SSRs from large-scale NGS sequence data, highlighting the critical needs and value of developing and deploying such strategies to bridge the knowledge gap.

**Table 1:** Bioinformatics tools developed based on Big Data technologies for handling large-scale sequence datasets.

**Big Data technologies**	**Tool**	**Year**	**Function**	**Software availability**	**Web server availability**
Hadoop	BigBWA (Abuín et al., 2015)	2015	Alignment	Yes	No
Spark	SparkBWA (Abuín et al., 2016)	2016		Yes	No
Spark	SparkSW (Zhao et al., 2015)	2015		Yes	No
Hadoop	Hadoop-BAM (Niemenmaa et al., 2012)	2012		Yes	No
Spark	DSA (Bo et al., 2017b)	2017		Yes	No
Spark	CloudSW (Bo et al., 2017a)	2017		Yes	No
Spark	SparkBLAST (Castro et al., 2017)	2017		Yes	No
Hadoop	Cloudblast (Matsunaga et al., 2009)	2008		Yes	No
Hadoop	HAlign (Zou et al., 2015)	2015		Yes	No
Hadoop	HSRA (Expósito et al., 2018)	2018		Yes	No
Spark	PASTASpark (Abuín et al., 2017)	2017		Yes	No
Hadoop	CloudAligner (Nguyen et al., 2011)	2011		Yes	Yes
Hadoop	CloudBurst (Schatz, 2009)	2009		Yes	No
Hadoop	BioPig (Nordberg et al., 2013)	2013	Sequence analysis	Yes	No
Hadoop	Halvade (Decap et al., 2015)	2015		Yes	No
Hadoop	Halvade-RNA (Decap et al., 2017)	2017		Yes	No
Spark	HiGene (Deng et al., 2016)	2016	Genome analysis	No	No
Spark	GATK-Spark (Li et al., 2017)	2016		No	No
Spark	SparkSeq (Wiewiórka et al., 2014)	2014		Yes	No
Hadoop	GATK (Mckenna et al., 2010)	2010		Yes	No
Spark	MEC (Zhao et al., 2017)	2017	Error correction	Yes	No
Hadoop	MarDRe (Expósito et al., 2017)	2017	Removal of duplicate DNA reads	Yes	No
Spark	MetaSpark(Zhou et al., 2017)	2017	Metagenomic read recruitment	Yes	No
Spark	Spaler (Abu-Doleh and Catalyurek, 2015)	2015	*De novo* genome assembly	No	No
Hadoop & Spark	SA-BR-MR and SA-BR-Spark (Dong et al., 2017)	2017	Sequence assembly	No	No
Hadoop & Spark	Falco (Yang et al., 2016)	2017	RNA-seq processing	Yes	No
Spark	SpaRC (Shi et al., 2018)	2019	Clustering analysis	Yes	No
Hadoop &Spark	GMQL (Masseroli et al., 2018)	2019	NGS tertiary data analysis	Yes	Yes
Hadoop	SeqPig (Schumacher et al., 2014)	2014	Sequence processing	Yes	No

Both Hadoop and Spark can deal with the above problems. Generally, Spark has better performances for iterative algorithms than Hadoop (Samadi et al., 2018). However, Spark is in-memory computing, and it becomes slower than Hadoop when the cluster has not enough memory. Thus, Hadoop is a better choice for the system without sufficient memory (Samadi et al., 2018). For merging paired-end reads and mining SSRs, we do not need to perform operations over the same data recursively and only need to choose Hadoop to address these two problems. In this work, we propose BigFiRSt (Big data-based Flash and peRf algorithm for mining Ssrs), a novel Hadoop-based program suite and is specifically designed to integrate paired-end reads merging and SSRs search into an effective computational pipeline. There are two fundamental modules in BigFiRSt: BigFLASH and BigPERF. They represent two implementations of the well-known stand-alone algorithms FLASH (Magoc and Salzberg, 2011) and PERF (Avvaru et al., 2017b) based on Hadoop techniques. Due to the advantages of the Hadoop big data technology, BigFLASH and BigPERF have significantly improved the computational efficiency compared with the baseline FLASH and PERF, respectively. Moreover, BigFiRSt allows users to apply BigFLASH and BigPERF separately and provides a pipeline functionality to enable users to run them consecutively. It allows the program to take short read pairs as the input and return the mined SSRs. These outputs can be used for genotyping analysis and other custom analyses (Budiš et al., 2018) to better suit users' specific needs.

Intuitively, it is more convenient for biologists to process and analyse large-scale sequences by a user-friendly web interface. However, in practice, it remains a challenging problem for users to upload large scale datasets from their local machines to the online web interface (Zou et al., 2014). To facilitate users to merge read pairs and subsequently identify SSRs in relatively small datasets, we provide a publicly available web interface of BigFiRSt, which is available at http://bigdata.biocie.cn/BigFiRSt/. There is no other such web interface integrating these two processes currently available in the research community to the best of our knowledge. On the other hand, for handling massive datasets and facilitating the data process using local computers, we also provide the source codes of BigFiRSt for download https://github.com/JinxiangChenHome/BigFiRSt such that users can configure and execute the BigFiRSt program on a cluster supported by the Hadoop.

## Design And Implementation

### Apache Hadoop

BigFiRSt was developed based on the Big Data Hadoop technology (White, 2009). Hadoop has been regarded as a milestone of big data processing (Petrillo et al., 2019). It is an open-source framework that can be installed on a Linux cluster for distributed processing of large-scale data sets using the MapReduce model (Dean and Ghemawat, 2008). MapReduce is a computation mode that allows users to specify a map and a reduce operation for parallelising the extensive computation. Generally, a Hadoop MapReduce job requires three core modules, namely, Hadoop Distributed File System (HDFS) (Shvachko et al., 2010), Hadoop MapReduce and Yet Another Resource Negotiator (YARN) (Vavilapalli et al., 2013). The input large-scale data sets are split into independent blocks and stored in HDFS across all Hadoop cluster computing nodes. Independent data blocks are processed by map tasks in a completely parallel manner. Reduce tasks fetch the corresponding partitioned data from the output of map tasks. YARN is responsible for resource management of the cluster and job scheduling/monitoring. Altogether, HDFS and YARN are able to provide the fault tolerance and data locality of Hadoop clusters (Taylor, 2010; Alnasir and Shanahan, 2018).

### Overview of the BigFiRSt Methodology

**Figure 1:**
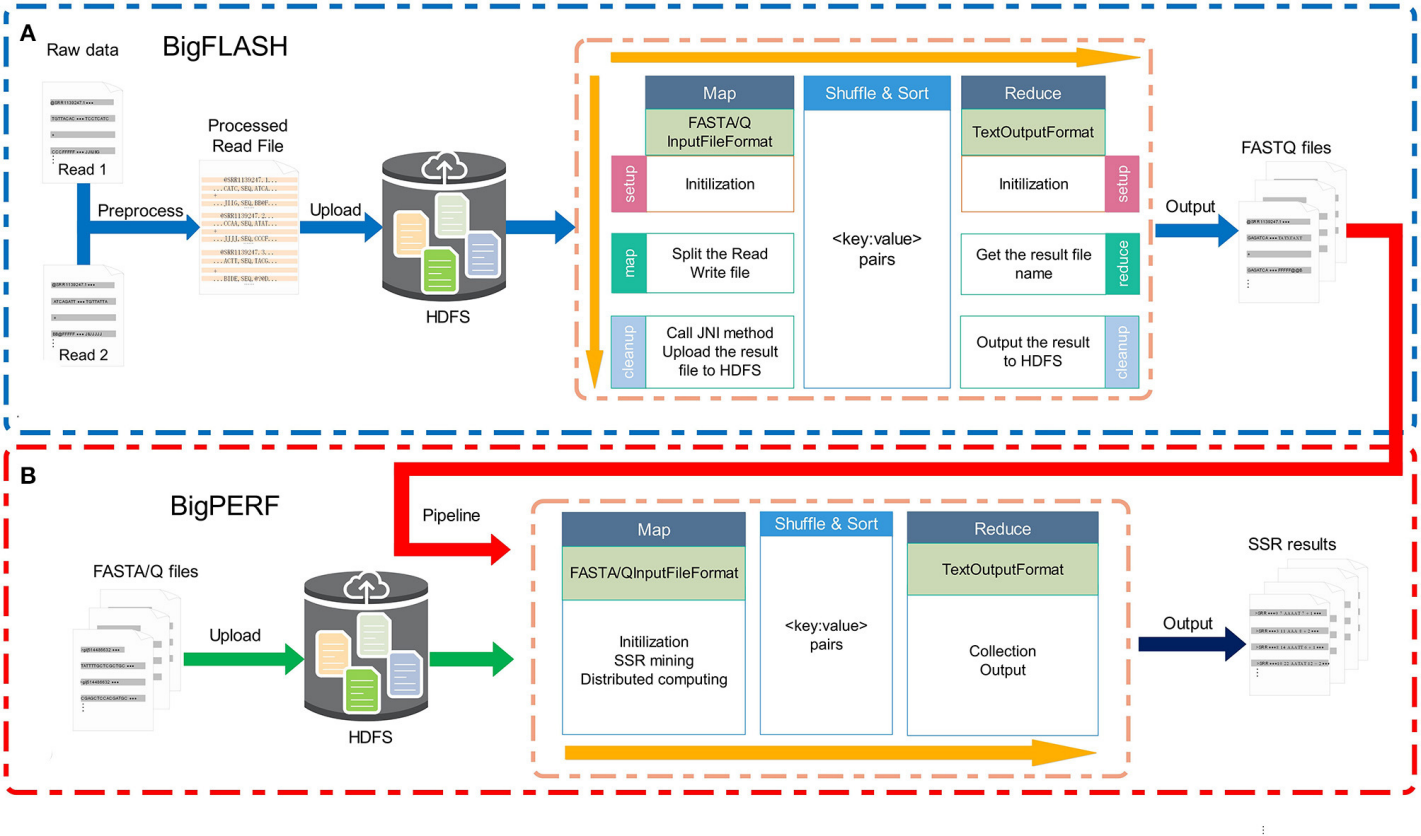
The overall framework of the BigFiRSt methodology. BigFiRSt contains two modules. **(A)** BigFLASH is used to merge short read pairs. **(B)** BigPERF is used to mine SSRs contained in reads.

The overall framework of the BigFiRSt methodology is illustrated in Figure 1. BigFiRSt contains two modules: BigFLASH (Figure 1A) and BigPERF (Figure 1B). BigFLASH is used to merge short read pairs and output long consensus reads, while BigPERF extracts SSRs from large-scale reads. These modules can be further integrated into a pipeline that takes the output of BigFLASH as the input to BigPERF. The red line in Figure 1 highlights the pipeline that connects BigFLASH with BigPERF.

### BigFiRSt

The Hadoop MapReduce module provides the Mapper interface with the Map method and the Reducer interface with the reduce method, respectively. A Hadoop application generally implements these two interfaces to create the map and reduce tasks. The number of map tasks depends on the number of InputSplits, which is a logical split of input files. InputSplits are created from data blocks, which exist physically on disk across Datanodes of clusters. In BigFiRSt, the size of InputSplit is the same as the block size, by default. The Hadoop MapReduce framework creates one map task to process each InputSplit in a completely parallel manner. Each InputSplit is generated by the InputFormat. In the Hadoop framework, FileInputFormat is the base class of all file-based InputFormat. The default InputFormat is TextInputFormat (a subclass of FileInputFormat), which breaks file into lines. The text of each line as value is processed by the map task. For BigFiRSt, the input data with the FASTQ format denotes a read for every four lines. The first line is the sequence title/identifier, which starts with a character “@.” The second line represents the nucleotide sequence of this read. The nucleotides in the sequence are usually presented in the upper case. The third line starts with “+” and contains a full repeat of the title line (the first line). The fourth line denotes the quality string of the sequence. Its length was equal to the sequence string (the second line). Hadoop cannot directly handle sequences with FASTQ format. We used a subclass of FileInputFormat written in (Ferraro Petrillo et al., 2017) to convert each InputSplit to a format that Hadoop can handle.

#### BigFLASH

BigFLASH implements the FLASH (Magoc and Salzberg, 2011) algorithm based on the Hadoop technology. FLASH has been extensively used for pre-processing large-scale NGS sequence data and facilitating the downstream analysis. Generally, it works by first merging read pairs into a consensus read preceding the analysis of SSR profiles based on NGS (van der Gaag et al., 2016; Hoogenboom et al., 2017; Ganschow et al., 2018). If cases where read pairs end within an SSR sequence, then the SSR sequence can be truncated after the read pairs are merged by FLASH. Accordingly, we used a Modified Version (1.2.11) of FLASH (2015) to implement BigFLASH.

The original FLASH algorithm was written in C programming language. However, Hadoop was programmed using the Java language and as such, it provides many useful Java APIs for Hadoop based application development. In general, Hadoop-based applications are implemented in Java in order to enable better interactions with Hadoop. Therefore, in BigFLASH, we used the Java Native Interface (JNI) (Liang, 1999) to integrate Java programming codes with the FLASH C code and effectively enable such interactions. This renders rewriting the source codes of FLASH unnecessary and ensures that no further modification of the original algorithm is required. We only used the FLASH source codes to build an additional shared library file named “libflash.so.” BigFLASH is able to parse the input parameters and then pass them on to the main method of FLASH by loading “libflash.so.”

**Figure 2:**
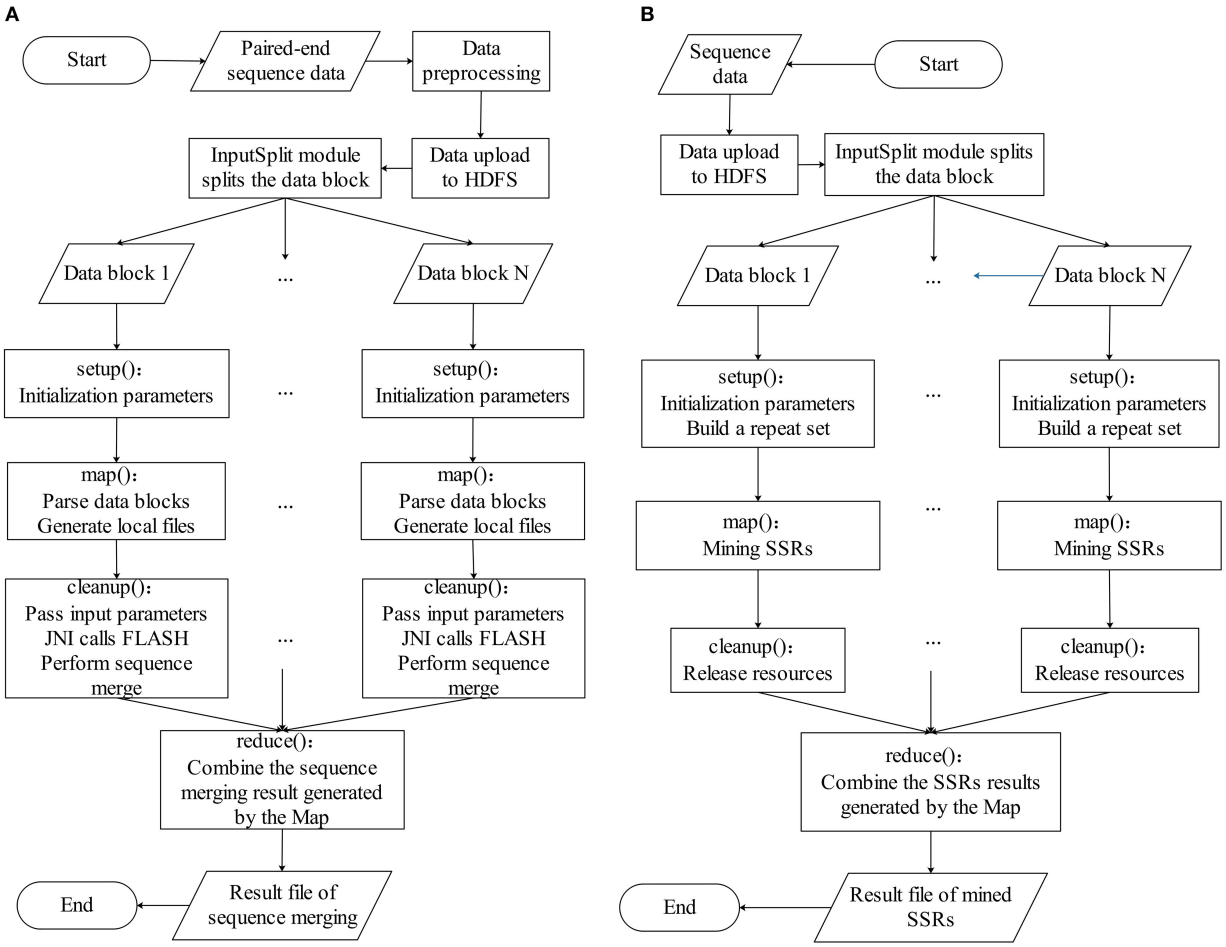
The detailed workflow of **(A)** BigFLASH and **(B)** BigPERF.

The BigFLASH process comprises of three major steps, which are illustrated in Figure 1A. The detailed workflow of BigFLASH is shown in Figure 2A. As can be seen, the first step is data pre-processing. The read pairs are stored in two separate FASTQ files. Considering that there is no API available in Hadoop for handling read pairs storing in two separate FASTQ files, we compiled a Python script (downloadable from the BigFiRSt web site) that can conveniently convert the two input FASTQ files into one single FASTQ file. The pseudo-code of the Python script is provided in the Supplementary Material. At the second step, the pre-processed data is uploaded to the HDFS, where large-scale data files are divided into fixed-size blocks. The third step is the MapReduce phase. BigFLASH applies the FASTAInputFileFormat/FASTQInputFileFormat function of FASTdoop (Ferraro Petrillo et al., 2017) to convert each data block to the Hadoop-acceptable format. Each block is processed by a Mapper. Each Mapper calls FLASH to merge the read pairs located in processed blocks, and all Mappers are executed in parallel. Lastly, the Reduce phase generates files of the merged reads by collecting the output of each Mapper. The key feature of BigFLASH is its Mappers, whose detailed procedures of implementation are described as follows: First, BigFLASH overrides the “setup” method from parent Class Mapper to parse the input parameters. The “setup” method will be invoked automatically, to initialize input parameters required by FLASH. Second, the “map” method from parent Class Mapper is overridden, to parse each InputSplits into two FASTQ files, which will be handled by FLASH. And finally, BigFLASH overrides the method “cleanup,” which passes the input parameters to the main method of FLASH by calling the declared native method.

#### BigPERF

As aforementioned, the original PERF program was written in Python (Avvaru et al., 2017b) and in this study, we rewrote it in Java to develop and implement BigPERF. The overall framework of BigPERF is shown in Figure 1B. The detailed workflow of BigFLASH is shown in Figure 2B. There exist three steps involved in the development of BigPERF. The first step is to upload the user input files in the FASTA/FASTQ format to HDFS. Similar to the second step in BigFLASH, the input data files are divided into fixed-size blocks and the FASTAInputFileFormat/FASTQInputFileFormat function is used to convert each block to a Hadoop-acceptable format. BigPERF first overrides the “setup” method from the parent Class Mapper to parse the input parameters and build a repeat set, which is then used for lookup during repeat identification. Then, the map method from the parent Class Mapper is overridden to mine all SSRs by extending the substrings appearing in the repeat set in a completely parallel manner. At this phase, each mapper generates a result file. The third step is the Reduce phase, where BigPERF collects the results from the map phase to generate the final results.

#### Reduce Operation

In the Reduce phase, BigFLASH and BigPERF only collect results from the output result files generated by each mapper. A complete Reduce task contains three primary phases: shuffle, sort and reduce. The Hadoop framework sorts the outputs of the mappers by keys simultaneously, and the shuffle phase fetches the relevant partitioned output of all mappers. Finally, the reduce phase calls the reduce method for each <key, (list of values)> pair in the grouped inputs.

Users can use the method “setNumReduceTasks” to set the number of reduce-tasks. A combined result output would be generated when the number of Reducers is set 1. The number of the result files depends on the predefined reducer number. Users are allowed to set the number of reduce-tasks to zero if no reduction is desirable. If the number of reduce-tasks is set to zero, the output of all the mappers is the result.

In BigFLASH and BigPERF, users have the option to select to use the “reduce” phase. In cases where such option is selected, according to the Hadoop MapReduce tutorial, the right number for “reduces” seems to be 0.95 or 1.75 multiplied by (<no. of nodes> ^*^ <no. of maximum containers per node>). The detailed information refers to the Hadoop tutorial on the official website.

### Web Server

We have implemented and deployed an online web server of BigFiRSt in order to facilitate users to merge read pairs and/or mine SSRs in small-scale datasets (up to 30 MB). The web server of BigFiRSt is freely accessible at http://bigdata.biocie.cn/BigFiRSt/. The two algorithms FLASH and PERF, as well as the computational pipeline have also been made available at this web server.

The BigFiRSt web server is managed by Tomcat 7 and hosted on a Linux server, equipped with a 1-core CPU, 40 GB hard disk and 2 GB memory. Using the web server, users can upload files, select desired parameters and obtain the result files.

#### FLASH

Users can merge paired-end reads by the FLASH algorithm via the web interface of BigFiRSt. This module works as follows: First, users need to upload both FASTQ format data files that respectively store the forward and reverse reads. Alternatively, users can also input the sequences in the FASTQ format in the text area. Second, users can update default parameter values of the FLASH algorithm using the web interface. Thirdly, users click to submit the job. Alternatively, users can also provide their email addresses in order to receive a notification Email after the submitted job is finished. Finally, when the submitted job is completed successfully, users can view the job details and download the generated results. In this case, users should have received such notification email and can check to review the job details by clicking a hyperlink in the email.

#### PERF

This module uses the PERF algorithm to mine SSRs from DNA sequences in FASTA format. Similar to using FLASH, users need to first upload sequence data and update default parameter values, and then submit this job. After the submitted job is completed, the user can view the detailed results of mined SSRs in a table. Moreover, users can input a preferred SSR and retrieve all reads containing this SSR from the result table. In addition, users can also export the mined results in the CSV/Excel format for the follow-up analysis in local computers.

#### Pipeline

The function of this module is to integrate FLASH with PERF into a pipeline. The pipeline first calls FLASH to merge read pairs of the input data, and then calls PERF to mine SSRs from the output of FLASH. Users only need to upload two FASTQ files containing pair-end reads or paste the data to this module, then update the default parameter value and submit the job, and finally obtain the result.

#### Other Functions

The web interface of BigFiRSt also provides other auxiliary functions including source code download, search for submitted jobs, view of all submitted jobs, and contact information, etc.

## Results

### Environment Setup

The experimental environment of BigFiRSt includes HDFS (version 2.7.3), YARN (version 2.7.3), MapReduce2 (version 2.7.3), Java (version 1.8), and Python (version 2.7.3).

**Figure 3:**
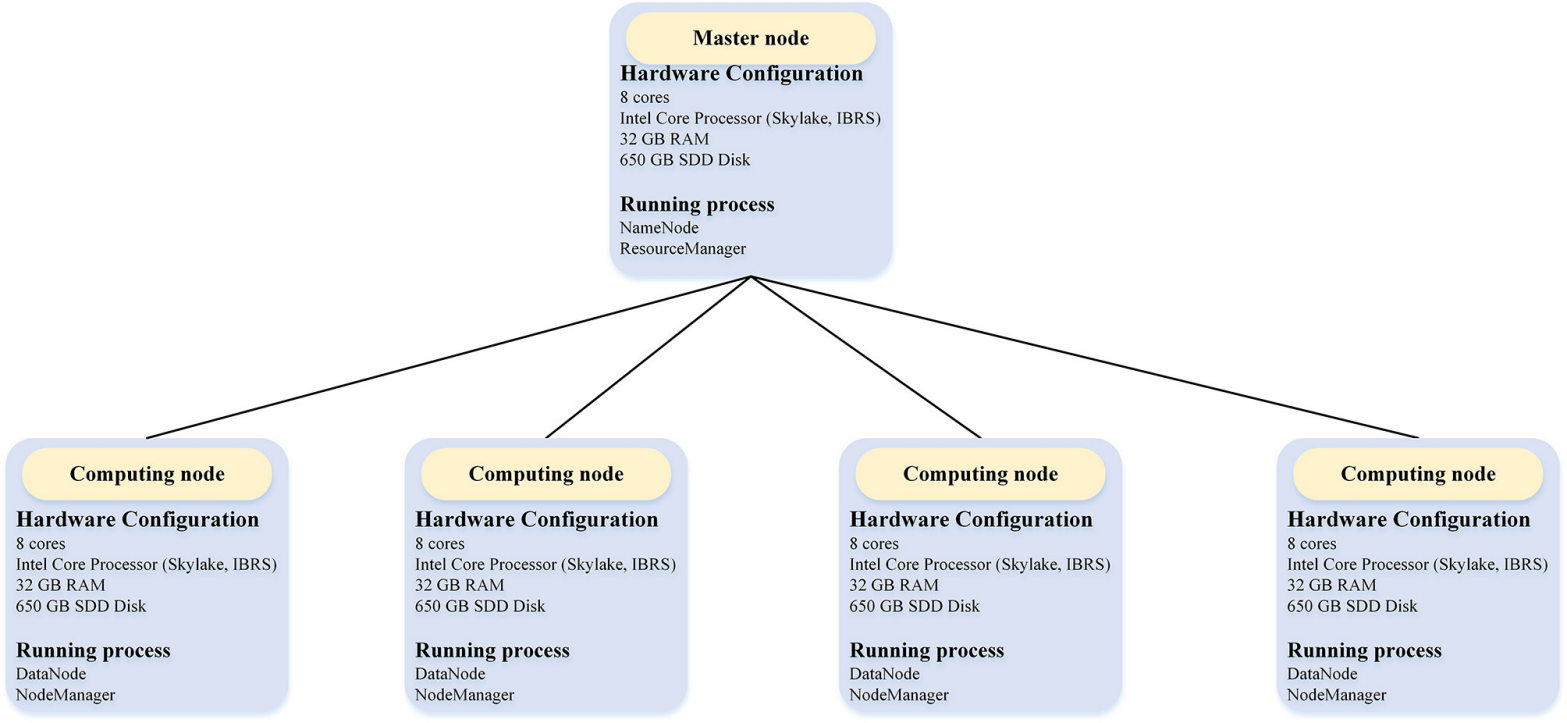
The overall architecture of the Hadoop cluster in the experiment.

We evaluated the performance of BigFiRSt using a five-node Hadoop cluster on the Research Cloud server of Monash University. The structure of this five-node Hadoop cluster with detailed hardware configurations is illustrated in Figure 3. One node of this cluster is a master node while the other four are computing nodes.

The master node is used for launching and managing the computational tasks, while four computing nodes are responsible for the Map/Reduce tasks. Table 2 provides the detailed information of the configuration of each machine used in the experiment. Each node had eight cores and 32 GB RAM memory. We configured the Hadoop “yarn-site.xml” file to allocate 4 GB memory for each of the eight cores for each node. Among these, seven cores were allocated to computational tasks and one core to the operating system. Accordingly, each node can run up to seven Map/Reduce tasks at the same time. That is, a total of 28 Map/Reduce tasks are allocated for four computing nodes. This arrangement also means that when we performed a 32-core experiment in a Hadoop cluster, we needed to set the master node as a compute node as well (i.e., using 4/8 cores of the master node for computing). The block size was set to 128 M by the Hadoop configure file.

**Table 2:** Configurations for each machine used in the experiment.

**Components**	**Configuration**	
	**Each node in cluster**	**Stand-alone node**
CPU in each node	Intel Core Processor (Skylake, IBRS)	Intel Core Processor (Skylake, IBRS)
The number of cores in each node	8	32
RAM Memory in each node	32 GB	128 GB
Disk in each node	650 GB SDD General Purpose disk	5 TB SDD General Purpose disk

For the performance comparison of BigFLASH, we conducted a comparative experiment in which the numbers of FLASH threads and Hadoop cluster cores were set as the same. Therefore, we added a 32-core stand-alone machine with the same hardware configurations as any of the machines in the cluster for experimentation (refer to Table 2 for more detail).

### Datasets

We employed three experimental datasets from The 1000 Genomes Project Consortium (2010) to examine the performance of BigFiRSt. A statistical summary of the three datasets used is provided in Table 3.

**Table 3:** Main characteristics of the input datasets for read pairs merging.

**Tag**	**Name**	**Total pairs**	**Read length (bp)**	**Size (GB)**
D1	SRR642648	99356100	100	52.2
D2	SRR642751	179922078	100	99.2
D3	SRR622459	1222689201	100	584.8

### Read Pair Merging

The main characteristics of the datasets used for the read pair merging phase is shown in Table 3. We compared the execution time between BigFLASH and FLASH for this process and obtained the performance results by averaging the execution time over the five experiments for each method. We ran FLASH five times on a 32-core stand-alone machine with the same hardware configurations as any of the machines in the cluster. All parameters used in the test were set as the default. The experimental results are shown in Table 4.

**Table 4:** Experimental results for merging read pairs by the original FLASH algorithm.

**Dataset**	**Average execution time (seconds)**				**Average number of pairs processed/second**				**Combined percent**
	**Number of threads**				**Number of threads**				
	**8**	**16**	**24**	**32**	**8**	**16**	**24**	**32**	
D1	1141.159	1036.956	1238.376	985.176	87,066	95,815	80,231	1,00,851	72.03%
D2	1579.457	1371.293	1431.130	1594.532	1,13,914	1,31,206	1,25,720	1,12,837	29.33%
D3	9821.888	9258.983	8867.265	9260.385	1,24,486	1,32,054	1,37,888	1,32,034	12.7%

Here the number of reduce-tasks was set to zero when running the BigFLASH. The average execution time for merging read pairs is shown in Table 5. We controlled the total number of cores in the cluster by modifying the mapred-site.xml and yarn-site.xml configuration files in Hadoop. The experimental results show that, for any one of the experimental datasets and as the number of cores in the cluster increases, the shorter the cluster execution time, in a roughly similar scale (as the number of splits in the dataset in Hadoop is greater than the number of cluster cores). As shown in Tables 4, 5, when employed 8 CPU cores cluster for BigFLASH, speedup ratios reach 2.630, 1.670, 1.832 on D1, D2, and D3, respectively. In comparison, when applied 32 CPU cores cluster, speedup ratios are improved to 5.950, 5.623, and 5.432 on D1, D2, and D3, respectively. In general, more CPU cores achieved more speedup ratios.

**Table 5:** Average execution time for merging read pairs by BigFLASH in the cluster.

**Dataset**	**Average execution time (seconds)**				**Speedup**			
	**Number of cores**				**Number of cores**			
	**8**	**16**	**24**	**32**	**8**	**16**	**24**	**32**
D1	433.835	293.052	200.535	165.563	2.630	3.538	6.175	5.950
D2	946.030	482.686	335.814	283.556	1.670	2.840	4.262	5.623
D3	5360.550	3162.039	2354.566	1704.687	1.832	2.928	3.766	5.432

On the other hand, we can also measure the performance of BigFLASH in terms of the number of read pairs processed per second in the Map phase. The sum of the execution time of all map tasks for each experiment is shown in Table 6. As can be seen, when using more cores in the cluster, the total running time of all Mappers would be more than those of all Mappers when less cores are used in the cluster. When more Mappers in each node would be running at the same time, each Mapper would be cost more times. However, more Mappers could be running at the same time when more cores are available in the cluster, thus it would cost less time to finish the map tasks (refer to Table 5).

**Table 6:** Execution time of all map tasks of BigFLASH in five experiments.

**Dataset**	**Number of cores**	**Execution time (seconds) of each experiment**				
		**1st**	**2nd**	**3rd**	**4th**	**5th**
D1	8	2857.762	2722.378	2644.078	2677.849	2626.778
	16	3808.937	3912.256	4098.451	3800.208	3839.815
	24	4254.219	3953.122	3927.272	3840.440	3914.564
	32	4347.978	4218.607	4292.595	4350.655	4452.450
D2	8	5894.834	6289.555	6053.989	6255.050	6087.408
	16	6625.868	6716.897	6722.830	6382.543	6497.228
	24	7225.822	6868.207	6736.306	6786.839	6720.426
	32	7775.674	7731.089	7676.768	7913.205	7626.669
D3	8	34644.069	33557.823	34702.026	36898.111	35523.228
	16	35653.879	45796.694	45262.908	44955.438	43740.268
	24	43463.962	42863.979	45251.582	57696.282	65181.182
	32	49702.159	49089.883	49223.694	48896.363	48600.491

Table 7 shows the average number of read pairs processed per second, which can be calculated as follows:


$$\begin{array}{l} avePairsPerSec=\frac{totalReadsNum}{aveExecutionTime\;}\times \left(numOfCores-1\right), \end{array}$$

where *avePairsPerSec* denotes the average number of read pairs processed per second, *totalReadsNum* is the total number of read pairs of the processed dataset, *aveExecutionTime* means the average execution time shown, while *numOfCores* denotes the number of cluster cores. *numOfCores* − 1 indicates that YARN's ApplicationMaster process occupied a single core for resource management and task monitoring and did not participate in calculations. From Table 7 we can find that BigFLASH can handle more pairs each second than FLASH and achieve the highest speed up rate 7.16. Taken together, we conclude that the performance of BigFLASH was considerably better than the original serial algorithm, greatly shortening the execution time of the original program and reducing the user waiting time.

**Table 7:** Amount of data processed in the Map phase of BigFLASH.

**Dataset**	**Average number of pairs processed/second**				**Speedup**			
	**Number of cores**				**Number of cores**			
	**8**	**16**	**24**	**32**	**8**	**16**	**24**	**32**
D1	2,57,041	3,82,931	5,74,468	7,10,922	2.952	3.997	7.160	7.049
D2	2,05,922	4,09,591	6,02,577	7,20,183	1.808	3.122	4.793	6.383
D3	2,44,084	4,25,709	5,52,586	7,71,923	1.961	3.224	4.007	5.846

### SSR Mining

The three datasets used for BigPERF and PERF performance evaluations in terms of SSRs mining are shown in Table 8. Note that these datasets were derived from the merged results of BigFLASH for the three datasets in Table 3.

**Table 8:** Input datasets for mining SSRs.

**Tag**	**Name**	**Total reads**	**Read length (bp)**	**Size (GB)**
D1‘	MSRR642648	71568961	100–200	14.4
D2‘	MSRR642751	52777550		12.1
D3‘	MSRR622459	155236691		30.4

We compared the execution time between PERF and BigPERF for the SSRs mining process. The resultant execution time on each dataset was obtained by averaging the time of the five randomized experiments. We randomly used one node of the cluster to run the original PERF algorithm five times on each dataset. The parameters used in the comparison experiments and the averaged running time (seconds) are shown in Table 9.

**Table 9:** Running information of PERF original algorithm.

**Dataset**	**Average execution time (seconds)**	**Average number of reads processed/second**	**Parameters**				
			**Cutoff**	**Min-motif-size**	**Parameters max-motif-size**	**Min-seq-length**	**Max-seq-length**
D1‘	16311.800	4,388	6	3	5	0	500
D2‘	12418.600	4,250					
D3‘	44976.800	3,451					

Here the number of reduce-tasks was set to zero when running the BigPERF. The execution time results are shown in Table 10. The sum of the execution time of all map tasks for each experiment is shown in Table 11, and the amounts of data processed by BigPERF in the Map phase are shown in Table 12, respectively. Similar to the experimental results for merging read pairs, the experimental results of SSR mining also exhibited consistent results. That is, the Hadoop-based algorithms (i.e., BigFLASH and BigPERF) are much more efficient compared with their original counterparts. Remarkably, we found that the performance improvement of BigPERF was extremely pronounced. For example, in terms of the execution time, BigPERF was at least 21 times faster (in the case of the D2‘ data set and the 8-core cluster) and at most 68 times (in the case of the D3‘ data set and the 32-core cluster) faster than that of PERF. In terms of the number of reads processed per second in the map phase, BigPERF runs at least 22 times faster (in the case of the D2‘ data set and the 8-core cluster) and at most 76 times faster (in the case of the D3‘ data set and the 32-core cluster) than PERF.

**Table 10:** Execution time of BigPERF for searching SSRs.

**Dataset**	**Execution time (seconds)**				**Speedup**			
	**Number of cores**				**Number of cores**			
	**8**	**16**	**24**	**32**	**8**	**16**	**24**	**32**
D1‘	718.982	437.236	319.176	273.860	22.687	37.307	51.106	59.562
D2‘	576.286	364.331	260.621	227.397	21.549	34.086	47.650	54.611
D3‘	1633.061	893.130	706.073	658.399	27.541	50.359	63.700	68.312

**Table 11:** Execution times of all map tasks of BigPERF in five experiments.

**Dataset**	**Number of cores**	**Execution time (seconds) of each experiment**				
		**1st**	**2nd**	**3rd**	**4th**	**5th**
D1‘	8	4714.821	4748.217	4734.551	4719.849	4705.533
	16	5695.032	6065.955	6049.859	6015.885	6108.858
	24	6568.444	6510.503	6434.264	6381.585	6398.347
	32	7350.969	7285.193	7149.244	7183.113	7102.169
D2‘	8	3823.328	3795.953	3752.347	3776.384	3752.877
	16	4816.881	4883.854	4815.179	4839.188	4855.779
	24	5351.771	5187.211	5184.548	5221.581	5214.872
	32	6055.070	5837.347	5917.858	5793.088	5863.227
D3‘	8	11080.473	11080.752	11109.434	11066.792	11126.353
	16	12746.333	12839.853	12649.824	12608.740	12767.178
	24	15301.268	15432.642	15299.017	15233.146	15261.768
	32	20687.485	19501.560	19232.093	17596.133	17472.989

**Table 12:** Amount of data processed by BigPERF in the Map phase.

**Dataset**	**Reads processed/second**				**Speedup**			
	**Number of cores**				**Number of cores**			
	**8**	**16**	**24**	**32**	**8**	**16**	**24**	**32**
D1‘	1,06,037	1,79,307	2,54,866	3,07,540	24.165	40.863	58.082	70.087
D2‘	97,732	1,63,493	2,32,012	2,77,620	22.996	38.469	54.591	65.322
D3‘	1,11,955	1,95,230	2,43,420	2,62,862	32.441	56.572	70.536	76.170

## Discussion

SSRs-pipeline (Miller et al., 2013) is a stand-alone tool that integrates read pairs merging and SSRs mining into a single pipeline. SSRs-pipeline first uses FLASH as a pre-processing algorithm of merging short read pairs from Illumina high-throughput DNA sequencing data and then employs a regular expression-based method to mine SSRs from merged read sequences. FLASH has been extensively used for pre-processing large-scale NGS sequence data and facilitating the downstream analysis (van der Gaag et al., 2016; Hoogenboom et al., 2017; Ganschow et al., 2018). Comprehensive experiments in Avvaru et al. (2017b) have shown that PERF is an extremely fast algorithm for mining SSRs. Moreover, PERF does not need to construct an extra complicated data structure for each read sequence. Thus, in this work, we selected FLASH and PERF to implement BigFiRSt using Big Data Technology. Obviously, there are many other well-known methods for mining SSRs and merging read pairs (review in the introduction section). The idea proposed in this paper can also be applied to implement other methods based on Big Data technologies.

**Figure 4:**
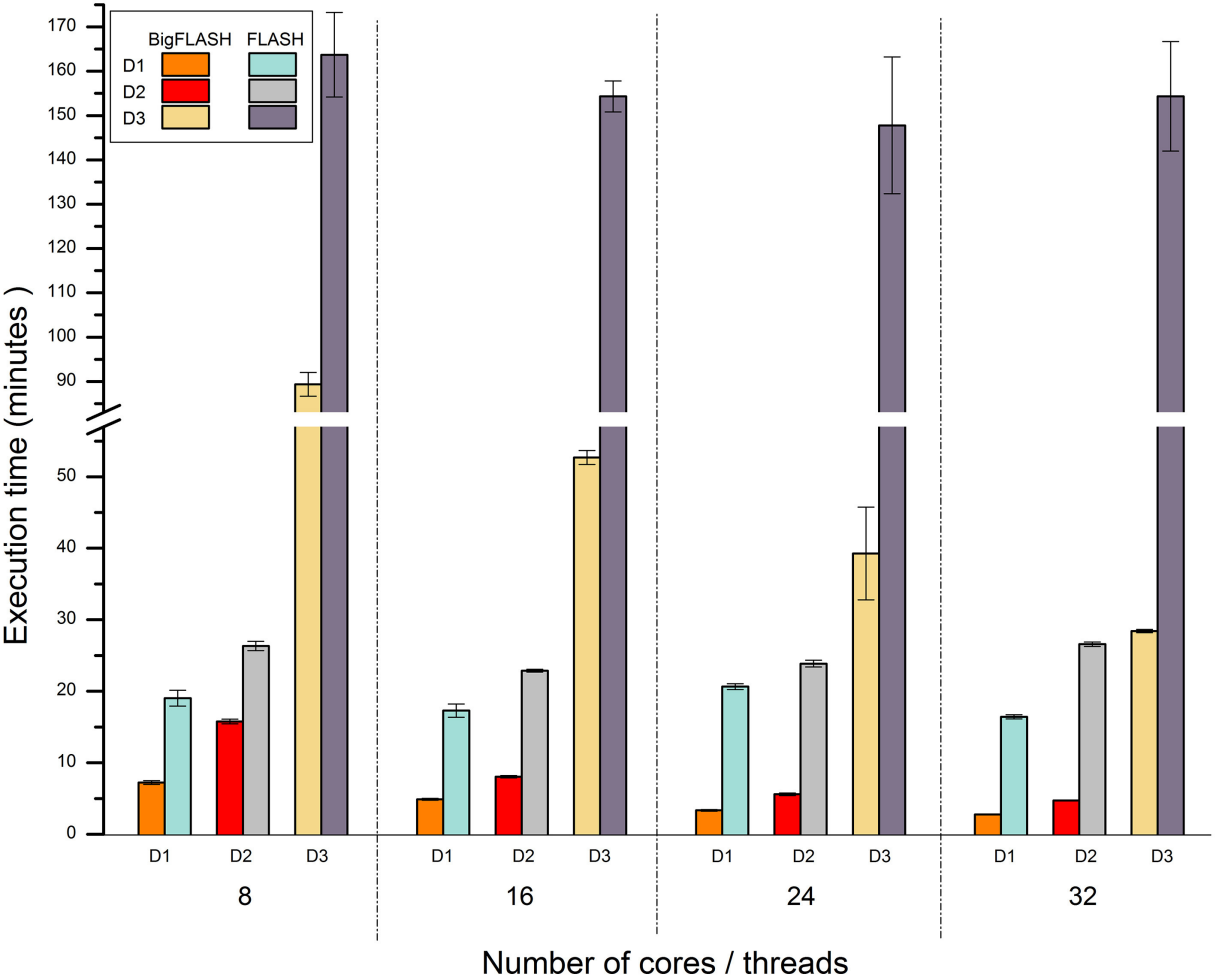
Runtime performance comparison between BigFLASH and FLASH for merging read pairs.

Currently, no published parallel methods for merging read pairs and mining SSRs are available. Thus, we only compared the performance of BigFLASH with FLASH, and that of BigPERF with PERF. Figure 4 illustrates the runtime performance comparison results between BigFLASH and FLASH. Although the original FLASH algorithm was a multithreaded algorithm with up to five threads, the execution time by FLASH was not apparently reduced as the number of the used cores increased. The reason is that only up to five threads could be used in FLASH. Thus, a supercomputer or cluster cannot further improve the performance of FLASH by simply adding more cores or more nodes. Compared with FLASH, BigFLASH significantly reduced the execution time for merging read pairs. For instance, FLASH had a running time of more than 2.46 h on the D3 dataset, which was more than 580 GB large. In contrast, BigFLASH (with 32 cores) only consumed 0.47 h to process the entire dataset. In addition, we can also see that the execution time was gradually reduced as the number of used cores increased, for each experimental dataset (across D1 to D3). Please refer to read pair merging for more detailed discussions.

**Figure 5:**
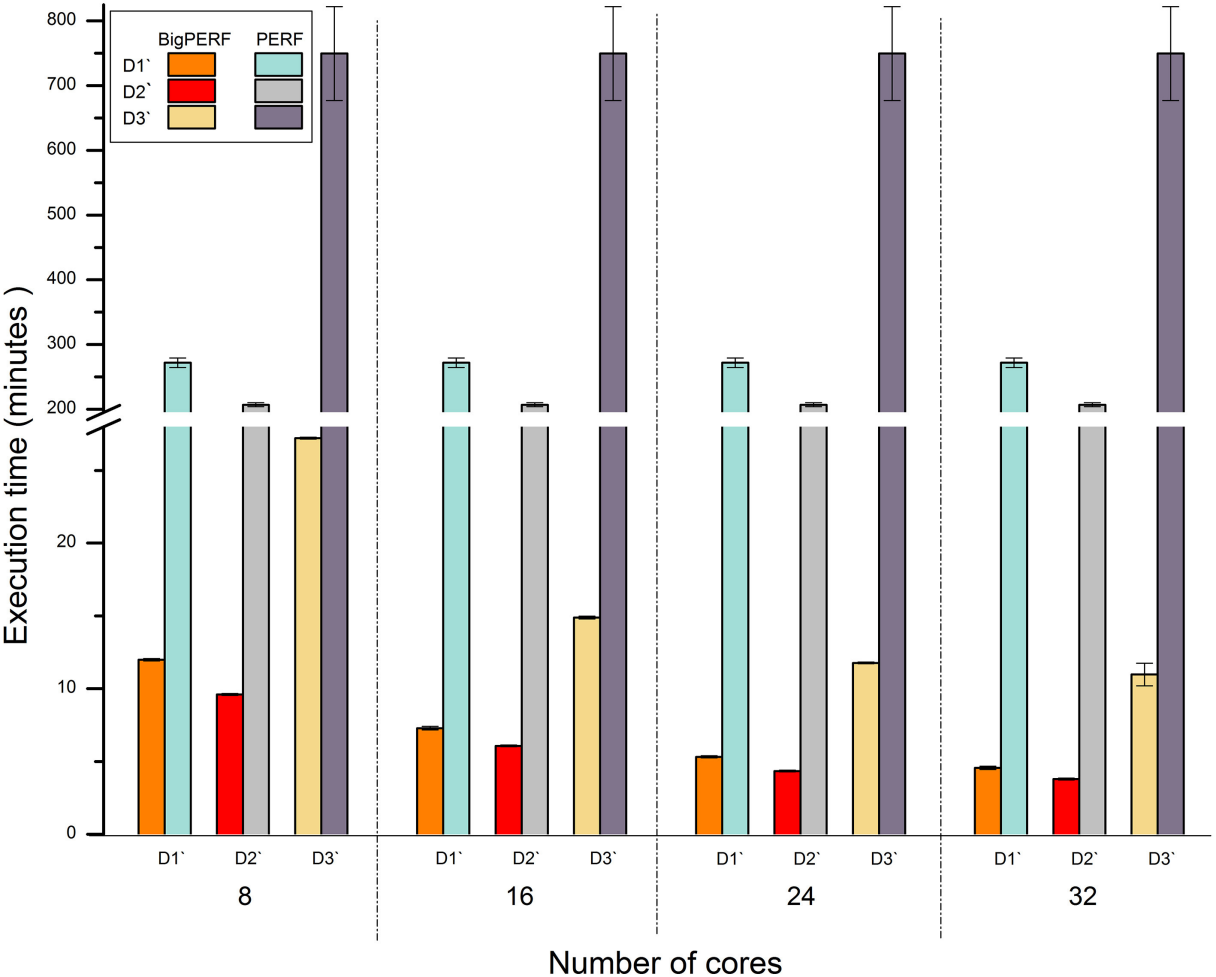
Runtime performance comparison between BigPERF and PERF for mining SSRs.

Figure 5 shows the performance comparison results between BigPERF and PERF. PERF required more than 12.49 h to process the D3' dataset using one node of our cluster, while it only took BigPERF (with 32 cores) 0.18 h to process the dataset on the same cluster. Similar to BigFLASH, the execution time of BigPERF could be gradually reduced for each experimental dataset with the increasing number of cores added. Please refer to SSR mining for more detailed discussions on this aspect.

### Limitations

Despite BigFiRSt improves the performance of the computational efficiency of read pair merging and SSR mining, it has the following limitations. It is great challenges for biologists to deploy a big data-based running environment. Although some commercial cloud-based big data platforms are available to run big data technique-based software, it remains a challenging problem for users to upload large scale datasets from their local machines to cloud platform. In addition, many large-scale datasets generated by NGS are costly, even some datasets may be private. Once datasets are uploaded to cloud platform, these datasets would be divulged. BigFiRSt have the above limitations. Thus, it is very interesting work to address the above issues for handling large scale sequences generated by NGS.

## Conclusion

There are two different types of *de novo* methods of SSRs identification, which mine SSRs from the entire genome and read sequences (Guo et al., 2018), respectively. The former heavily relies on high-quality entire genomes. It is practically very difficult to obtain a sufficiently good reference genome. Even for the human reference genome, several repeats may still be missing (Chu et al., 2016). In this scenario, it is beneficial for the latter to directly mine SSRs from large-scale sequencing reads generated by NGS techniques. While sequence reads generated by NGS are representative big data, conventional stand-alone methods often suffer from computational bottlenecks.

Thus, in this work, we have developed a program suite termed BigFiRSt based on the Big Data Hadoop technology to address the critical need of efficiently mining SSRs from large-scale NGS sequence datasets. For long enough reads produced by third-generation sequencing (e.g., Nanopore, PacBio), we need only use BigPERF (one module of BigFiRSt) to search SSRs contained in reads. For the short length of paired-end reads generated by second-generation sequencing (e.g., Illumina, SOLiD, IonTorrent), we can use the pipeline of BigFiRSt to first merge overlapping read pairs and then mine SSRs contained in merged read sequences. Alternatively, we used BigFLASH (another module of BigFiRSt) as pre-processing to merge read pairs into consensus sequences for other downstream analyses. Extensive benchmarking tests have shown that BigFiRSt has significantly improved the computational efficiency when merging read pairs and mining SSRs from the large-scale datasets. In the future era of big data, especially given the development of new sequencing techniques and rapid generation of sequence data, we anticipate that BigFiRSt will prove to be a valuable tool.

## Data Availability Statement

The original contributions presented in the study are included in the article/Supplementary Material, further inquiries can be directed to the corresponding authors.

## Author Contributions

JS and QL conceived the initial idea and designed the methodology. JC and FL implemented the experiments and processed the results. All authors drafted, revised, and approved the final manuscript.

## Funding

QL was supported by the grant from National Natural Science Foundation of China (61972322), the Provincial Natural Science Foundation of Shaanxi Province (2021JM-110). JS was supported by grants from the National Health and Medical Research Council of Australia (NHMRC) (APP490989, APP1127948, and APP1144652), the Australian Research Council (ARC) (LP110200333 and DP120104460), and the National Institute of Allergy and Infectious Diseases of the National Institutes of Health (R01 AI111965).

## Conflict of Interest

The authors declare that the research was conducted in the absence of any commercial or financial relationships that could be construed as a potential conflict of interest.

## Publisher's Note

All claims expressed in this article are solely those of the authors and do not necessarily represent those of their affiliated organizations, or those of the publisher, the editors and the reviewers. Any product that may be evaluated in this article, or claim that may be made by its manufacturer, is not guaranteed or endorsed by the publisher.

## Abbreviations

SSRs: Simple Sequence Repeats
NGS: next-generation sequencing
STRs: short tandem repeats
BigFiRSt: Big data-based Flash and peRf algorithm for mining Ssrs
HDFS: Hadoop Distributed File System
YARN: Yet Another Resource Negotiator.
