# Supplementary material for: BigFiRSt: A Software Program Using Big Data Technique for Mining Simple Sequence Repeats From Large-Scale Sequencing Data
Source: Front Big Data. 2022 Jan 18;4:727216. doi: 10.3389/fdata.2021.727216 (PMC8805145; doi:10.3389/fdata.2021.727216)
Supplement: Supplementary file 1 [file Data_Sheet_1.docx]

**BigFiRSt: a software program using big data technique** **for mining simple sequence repeats** **from large-scale sequencing data**

Jinxiang Chen^1, †^, Fuyi Li^2,3,4 †^, Miao Wang^1^, Junlong Li^1^, Tatiana T. Marquez-Lago^5^, André Leier^5^, Jerico Revote^2^, Shuqin Li^1^, Quanzhong Liu^1,*^, Jiangning Song^2,3,*^

^1^College of Information Engineering, Northwest A&F University, Yangling 712100, China;

^2^Biomedicine Discovery Institute and Department of Biochemistry and Molecular Biology, Monash University, Melbourne, VIC 3800, Australia;

^3^Monash Centre for Data Science, Monash University, Melbourne, VIC 3800, Australia;

^4^The Peter Doherty Institute for Infection and Immunity, The University of Melbourne, VIC, Australia;

^5^Department of Genetics and Department of Cell, Developmental and Integrative Biology, School of Medicine, University of Alabama at Birmingham, AL, USA.

^†^The first two authors contributed equally to this work.

^*^To whom correspondence should be addressed: (1) Jiangning Song, Email: [Jiangning.Song@monash.edu](mailto:Jiangning.Song@monash.edu); (2) Quanzhong Liu, Email: [liuqzhong@nwsuaf.edu.cn](mailto:liuqzhong@nwsuaf.edu.cn).

1. **BigFiRSt manual**

**1.1 Cluster Management**

Our cluster is managed by Apache Ambari. The web interface of Apache Ambari is shown in **Figure S1**. We can perform a series of operations to the cluster through Ambari web interface, such as cluster restart, parameter configuration, etc. Please refer to the steps given by Hadoop official website^[[1]](#footnote-1)^ for more details about the set-up of a cluster.


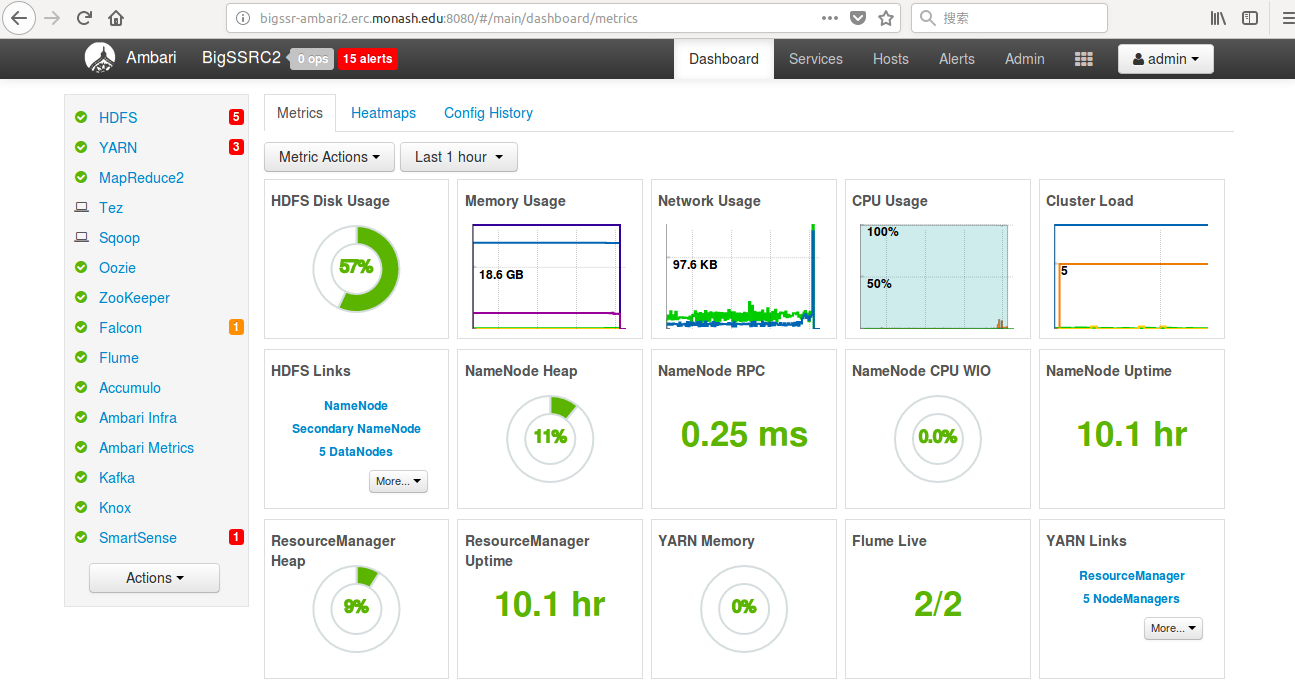


**Figure S1**. The web interface of Apache Ambari for managing and monitoring clusters.

## 1.2 Usage of BigFiRSt

After downloading the source code from the link provided in our article and unzipping it, users will see the following information (**Figure S2**).


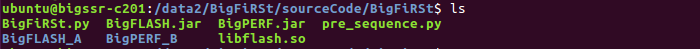


**Figure S2.** Source code package of BigFiRSt

where “BigFLASH_A” and “BigPERF_B” folders contain the source code of BigFLASH and BigPERF, respectively.

“pre_sequence.py” is used for the data preprocessing, it can merge two FASTQ files into one FASTQ file. Detailed description of the merging process is shown in **Section 1.2.2**.

“BigFLASH.jar” and “BigPERF.jar” files are two Java Archive files (jar) which were packaged based on the source code of BigFLASH and BigPERF. Users can directly deploy these two jar files to the Hadoop cluster to run the BigFiRSt. The detailed explanation of the usage to these two files is shown in **Sections 1.2.2, 1.2.3** and **1.2.4**.

“libflash.so” is a dynamic link library which was generated for running FLASH algorithm. If the users want to run BigFiRSt by using the jar file we provided, it doesn't need to recompile this dynamic link library, just follow the steps in **Sections 1.2.2, 1.2.3 and 1.2.4.** However, if the users need to change the source code and implement other function by themselves, they need to recompile the dynamic link library. The detailed description about how to compile this dynamic link library is shown in **Section 1.3**.

“BigFiRSt.py” file is the entry to the main function of the BigFiRSt pipeline. The users only need to run this file to freely choose the functions they need. This file needs to be used in conjunction with other files in the archive. The detailed explanation of its usage is shown in the following **Section 1.2.1**.

**1.2.1 Parameter description**

All parameters of BigFiRSt are shown in **Figure S3**. These parameters are divided into three types, which will be referred to as module parameters, BigFLASH optional parameters and BigPERF optional parameters. Module parameters are required when users run BigFLASH or BigPERF separately, as well as when running an automatics pipeline (BigFiRSt). The details of module parameters are shown **Figure S4.**

When users run BigFLASH separately, “-bf”, “-hh”, “-mH”, “-cP”, “-fj” and “-fmc” must be specified. Then, "BigFiRSt.py" will automatically parse these parameters. Among the given parameters, the parameter “-bf” is selected for running BigFLASH, “-hh” indicates the path to the installation directory of Hadoop, “-mH” represents the host name of the master node in the Hadoop cluster, “-cP” stands for rpc port of hdfs in Hadoop cluster, “-fj” is selected for using the Jar package “BigFlash.jar”, and “-fmc” means which is the main class (entrance) in the “BigFLASH.jar”.

In the same way, when users run BigPERF separately, “-bp”, “hh”, “-mH”, “-cP”, “-pj” and “-pmc” are required parameters. When running an automatic pipeline of BigFiRSt, “-bfp”, “-hh”, “-mH”, “-cP”, “-fj”, “-fmc”, “-pj”, and “-pmc” are selected. For a more detailed explanation of use of BigFiRSt, please refer to **Sections 1.2.2, 1.2.3** and **1.2.4**.

| BigFiRSt$ python BigFiRSt.py -h |
| --- |

**
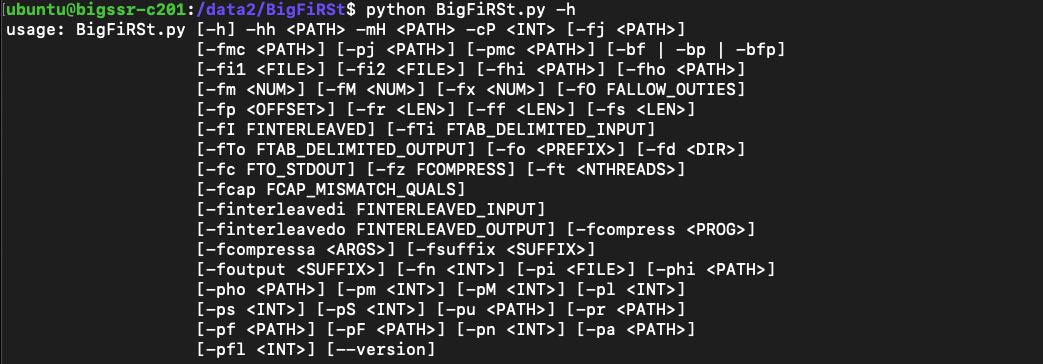
**

**Figure S3.** All parameters of BigFiRSt


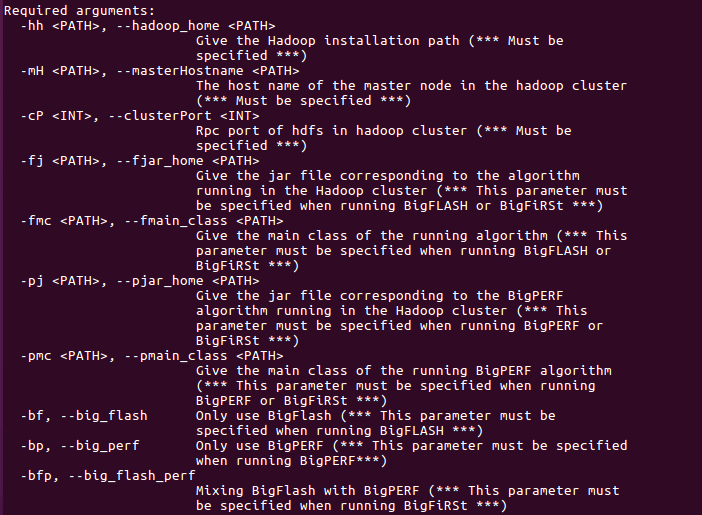


**Figure S4.** Module parameters of BigFiRSt

**Figure S5** shows the parameters used for running BigFLASH to merge read pairs, including “-fi1”, “-fi2”, “-fhi”, “-fho”, “-fm”, “-fM”, etc. Among these parameters, when we run BigFLASH, users must specify "-fi1", “-fi2”, “-fhi” and “-fho”. The “-fi1” and “fi2” parameters represent two FASTQ files storing the forward and reverse read pairs respectively, “-fhi” indicates the input path of BigFLASH in HDFS, “-fho” represents the output path of BigFLASH in HDFS, “-fm” means that the minimum overlap length between two reads that will be merged and the default value is 10 bp, and “-fM” represents that the maximum overlap length between two reads that will be merged and the default value is 65 bp.

**Figure S6** shows the parameters required for running BigPERF for mining SSRs, including “-pi”, “-phi”, “-pho”, “-pm”, “-pM”, etc. Among these parameters, when users run BigPERF, they must specify "-pi", “-phi” and “-pho”. The parameter “-pi” represents the input file, “-phi” indicates the input path of BigPERF in HDFS, “-pho” represents the output path of BigPERF in HDFS, “-pm” indicates the minimum size of the motif to be mined in the sequence, with a default value of 3, and “-pM” represents that the Maximum size of the motif to be mined in the sequence, with a default value of 5.


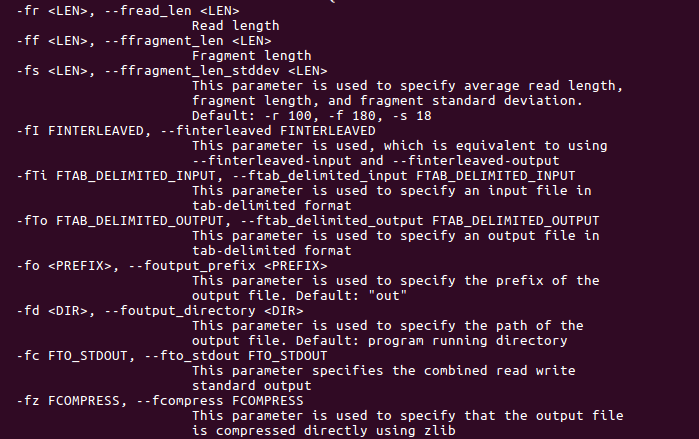

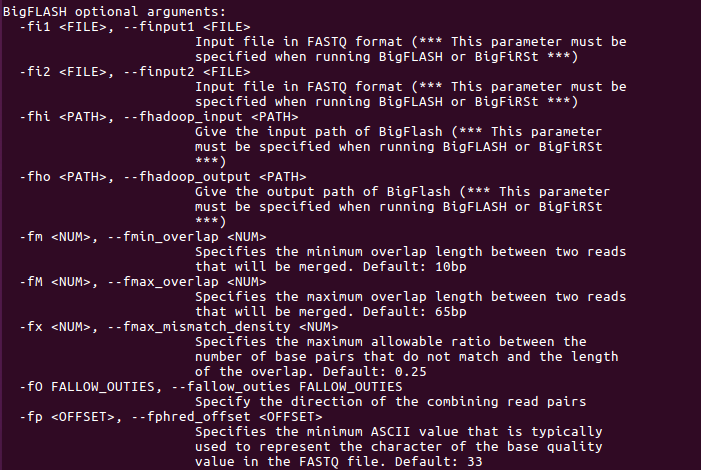


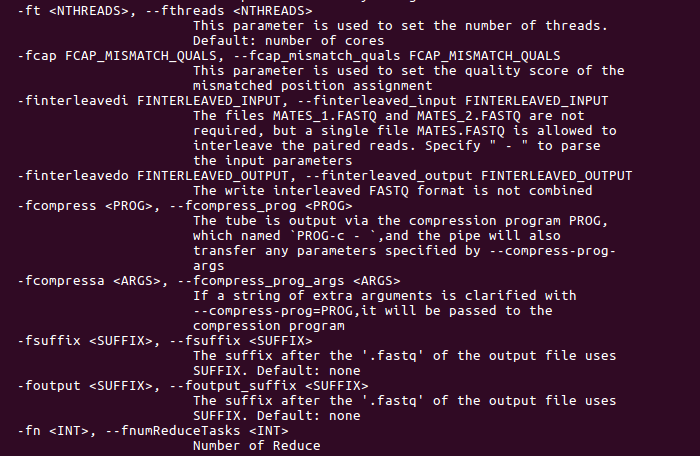


**Figure S5.** Algorithm parameters of BigFLASH

**
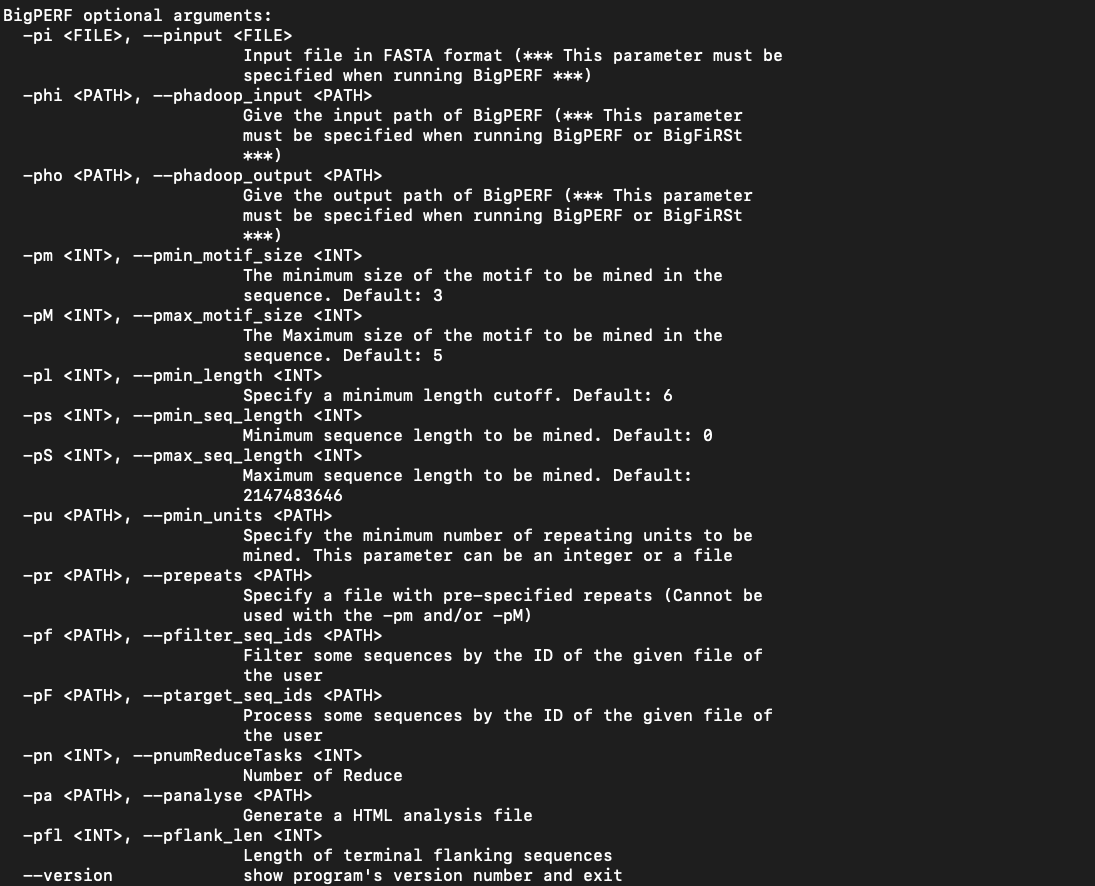
**

**Figure S6.** Algorithm parameters of BigPERF

**1.2.2 BigFiRSt usage**

The code “BigFiRSt.py” in the source project is used for running the pipeline of BigFiRSt. A detailed description of the pipeline is shown below (**Step 1 to 4**).

**Step 1**: preprocessing of read pairs. For big data analysis, large-scale data files are divided into fixed-size “chunks” [1]. Each read pair must be partitioned into the same “chunk” for correctly merging them into a single composite DNA sequence. However, the read pairs are usually stored in two FASTQ files. Therefore, in the BigFiRSt pipeline, we have to merge read pairs into one FASTQ file. An example of read pairs is shown in **Figure S7**. The python script “pre_sequence.py” provided in the source project is used for preprocessing input data.

**Figure S7**. Preprocess read pairs

The pseudo-code of the python script is described as follows.

| **Algorithm 1** Converting two FASTQ files into one FASTQ file |
| --- |
| **Input:** |
| Paired-end sequencing data, R1; |
| Paired-end sequencing data, R2; |
| **Output:** |
| Processed read file, PR. |
| **Begin:** |
| 1: Set *i*=1; |
| 2: **for (**line **in** R1**)** **do** |
| 3: line2 = *next*(R2) |
| 4: **if**(*i* % 4 != 2 **and** *i* % 4 != 0) **then** |
| 5: PR.write(line) |
| 6: **else** |
| 7: PR.write(line+",SEQ,"+line2) |
| 8: **end if** |
| 9: *i* =*i* + 1 |
| 10: **end for** |
| **End** |

**Step 2:** uploading preprocessed data into HDFS.

**Step 3:** merging read pairs. BigFLASH is called to merge read pairs into single composite DNA sequences. The result files are stored in HDFS.

**Step 4:** mining SSRs. BigPERF is called to analyze the result files generated by **Step 3,** to mine SSRs.

In what follows (**Step 1 to 5**), we provide an example to show how BigFiRSt combines BigFLASH and BigPERF into the pipeline:

| BigFiRSt$ sudo axel [ftp://ftp.sra.ebi.ac.uk/vol1/fastq/SRR642/SRR642751/SRR6427-51_1.fastq.gz](ftp://ftp.sra.ebi.ac.uk/vol1/fastq/SRR642/SRR642751/SRR642751_1.fastq.gz) |
| --- |

**Step 1:** Download and unzip the experiment input files from the 1000 Genomes Project [2]. The details are as follows:


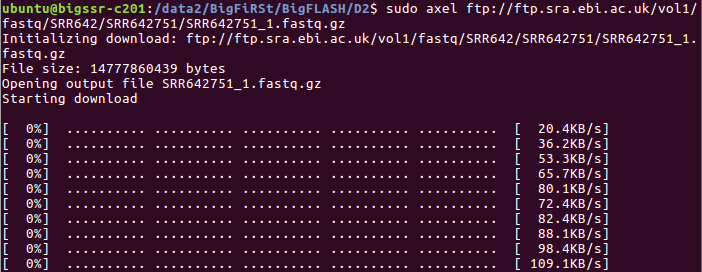


| BigFiRSt$ sudo axel [ftp://ftp.sra.ebi.ac.uk/vol1/fastq/SRR642/SRR642751/SRR6427-51_2.fastq.gz](ftp://ftp.sra.ebi.ac.uk/vol1/fastq/SRR642/SRR642751/SRR642751_2.fastq.gz) |
| --- |


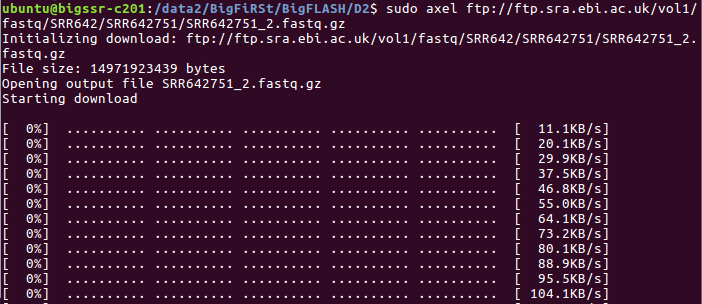


Unzip the downloaded files.

| BigFiRSt$ gunzip SRR642751_1.fastq.gz -c > ./SRR642751_1.fastq  BigFiRSt$ gunzip SRR642751_2.fastq.gz -c > ./SRR642751_2.fastq |
| --- |

**Step 2:** Initialize the Hadoop cluster. Before running BigFiRSt, users must ensure that the HDFS of the Hadoop cluster and the machine running “BigFiRSt.py” have enough space to store the preprocessed data. In addition, users need to distribute the pre-generated dynamic link library file (“libflash.so”) to each node (computer) in the cluster to the specified location (“/data2/BigFiRSt”). The details are as follows:


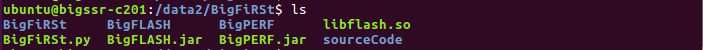


**Step 3:** Change the owner of Hadoop cluster to “**hdfs**” users:

| BigFiRSt$ sudo su hdfs |
| --- |


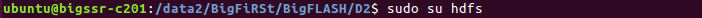


**Step 4:** Create a working directory in HDFS. The details are as follows:

| BigFiRSt$ Hadoop fs -mkdir -p /BigFiRSt/pipeline/input |
| --- |


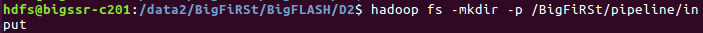


**Step 5:** Run BigFiRSt pipeline on the Hadoop cluster. The execution command of BigFiRSt is as follows:

| BigFiRSt$ python BigFiRSt.py \  -bfp \  -hh /data2/Hadoop \  -mH bigssr-c201.erc.monash.edu \  -cP 8020 \  -fj /data2/BigFiRSt/BigFLASH.jar \  -fmc com/nwafu/cjx/bigflash/BigFlashRunner \  -fi1 /data2/BigFiRSt/BigFLASH/D2/SRR642751_1.fastq \  -fi2 /data2/BigFiRSt/BigFLASH/D2/SRR642751_2.fastq \  -fhi hdfs://bigssr-c201.erc.monash.edu:8020/BigFiRSt/pipeline/input \  -fho hdfs://bigssr-c201.erc.monash.edu:8020/BigFiRSt/pipeline/output \  -pj /data2/BigFiRSt/BigPERF.jar \  -pmc nwafu/cie/bigssr/BigPerfRunner2 \  -phi hdfs://bigssr-c201.erc.monash.edu:8020/BigFiRSt/pipeline/output \  -pho hdfs://bigssr-c201.erc.monash.edu:8020/BigFiRSt/pipeline/output/output_perf \  -pm 3 -pM 5 -pl 6 -ps 0 -pS 500 |
| --- |

The BigFiRSt pipeline automatically conducts a series of operations, such as data pre-processing, uploading to HDFS, distributed computing, merge read pairs, and mining SSRs. Users only need to wait for the execution results. The details of the BigFiRSt pipeline running in the Hadoop cluster are shown in **Figure S8** and **Figure S9**. The specific usage of the BigFiRSt parameters is described in **Section 1.2.1**.


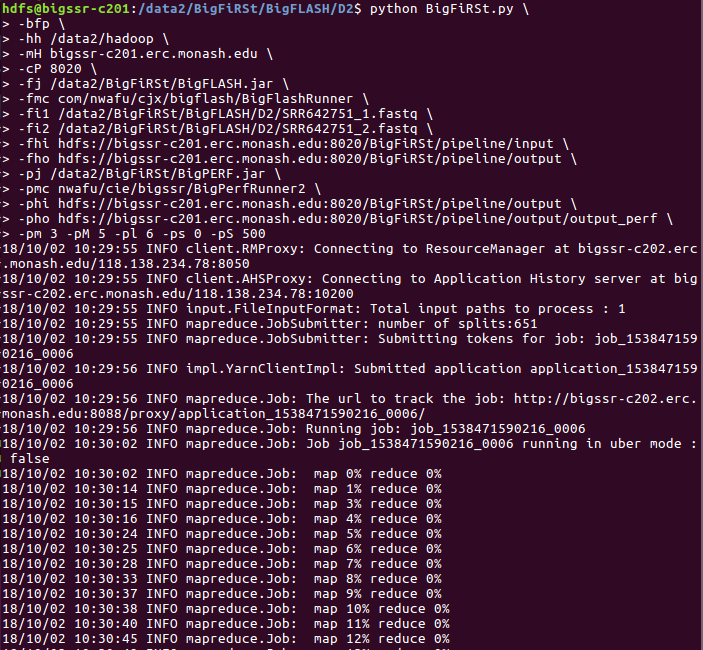

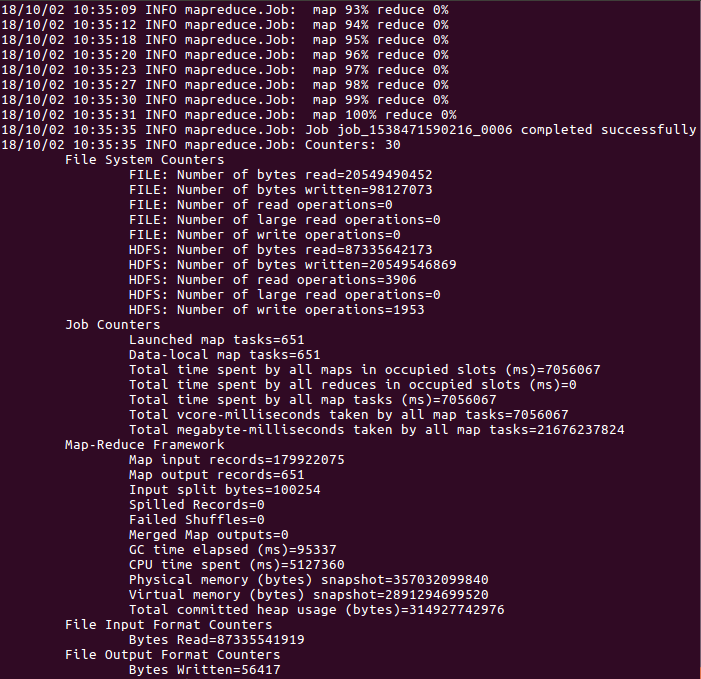


**Figure S8**. BigFLASH module running in BigFiRSt pipeline


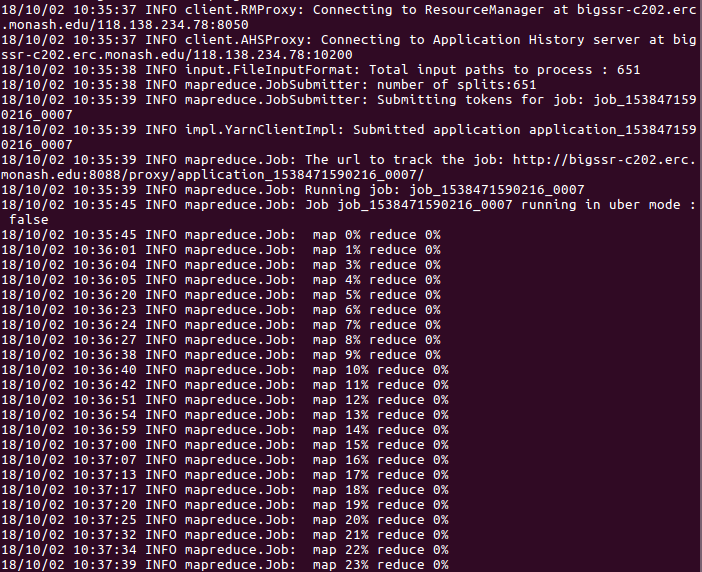


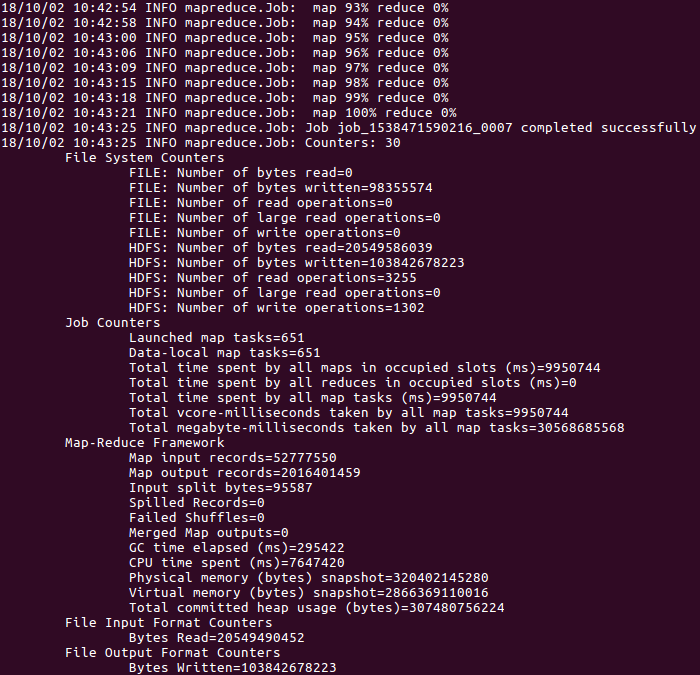


**Figure S9**. BigPERF module running in BigFiRSt pipeline

While running BigFiRSt tasks, users can utilize the 8088 port of the Hadoop web interface to check the HDFS and the running status of the Hadoop cluster. This web interface enables the details of running tasks, in real time. When the tasks are completed, users can download the results from HDFS. The web interface of Hadoop 8088 port is shown in **Figure S10**.


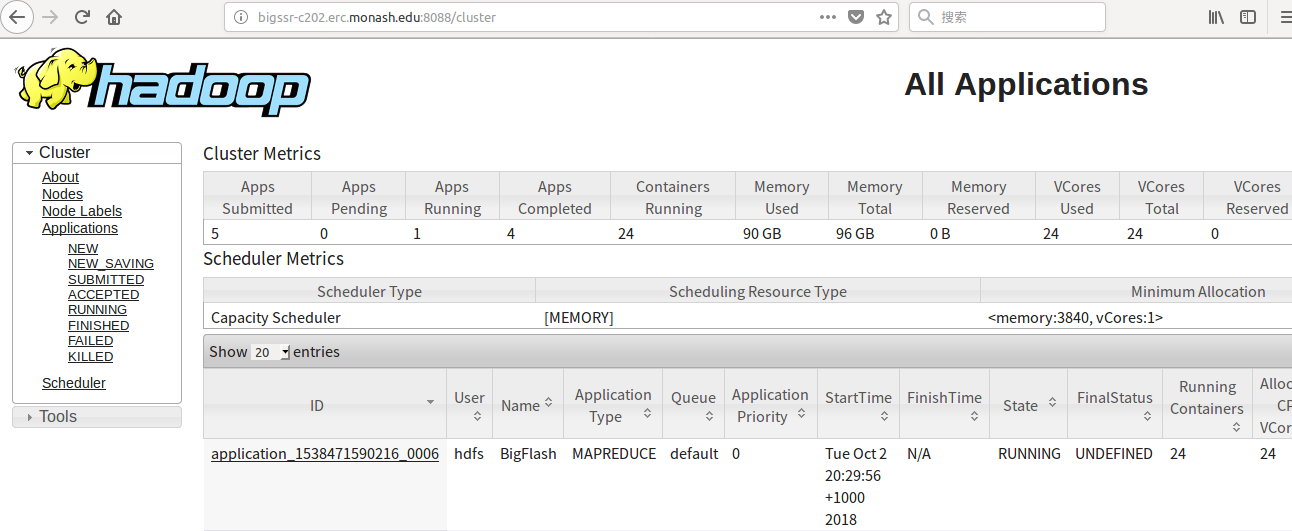


**Figure S10**. The web interface of Hadoop 8088 port

From the Hadoop 8088 port web interface, users can find that the cluster is running on 24 containers simultaneously. The memory of the cluster is set to 96GB and 24 VCores, and the cluster uses 90GB RAM and 24 VCores. In addition, the running status of each container can be viewed through the web interface. Users can maximize the advantages of the Hadoop cluster by modifying the parameters of the Hadoop configuration files [mapred-site.xml](http://hadoop.apache.org/docs/stable/hadoop-mapreduce-client/hadoop-mapreduce-client-core/mapred-default.xml) and [yarn-site.xml](http://hadoop.apache.org/docs/stable/hadoop-yarn/hadoop-yarn-common/yarn-default.xml) when conduct the experiments.

Users can also view the BigFiRSt working directory using the 50070 Web port of the Hadoop web interface. Details are shown in **Figure S11**.


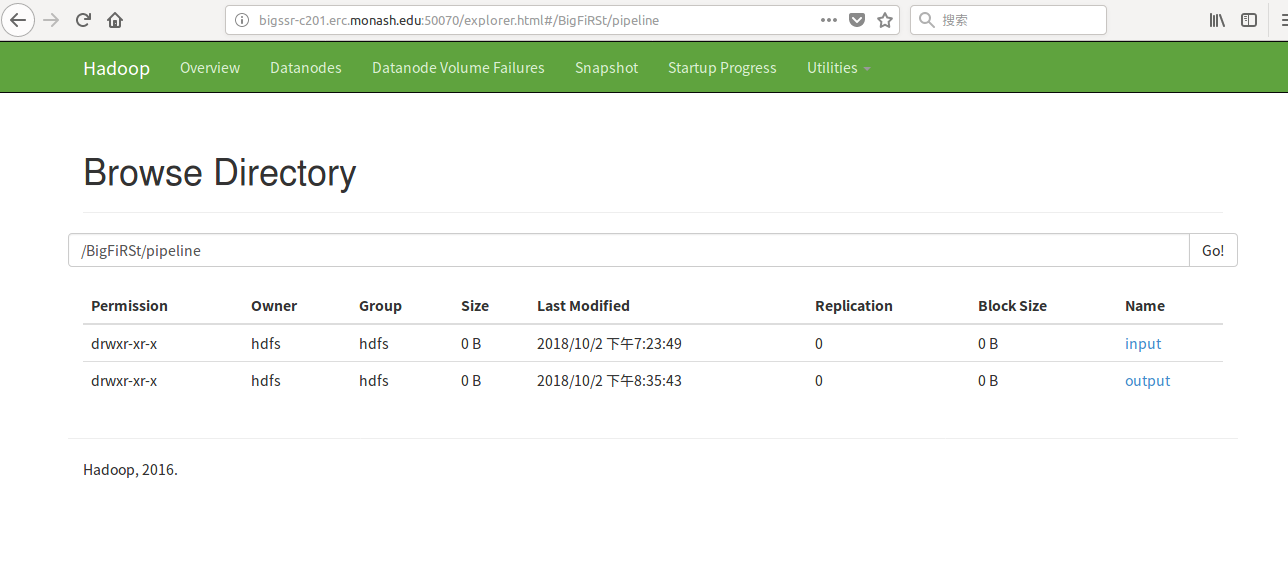


**Figure S11**. The web interface of Hadoop 50070 port

The output directory of BigFiRSt is shown in **Figure S12**, where the “file” folder and the “part-m-0000*” file are the output of BigFLASH. The “file” folder is specified with the parameter “-fho”, and the merged result files are located in this folder. The “part-m-0000*” file is an index file of the BigFLASH running result. The “output_perf” folder is the output of BigPERF. If the experiment settings have zero under “reduce number”, please refer to the description of BigFLASH in this section. However, if the reduce number of BigFLASH is not set to zero, please refer to the description of BigFLASH in **Section 1.2.3**.


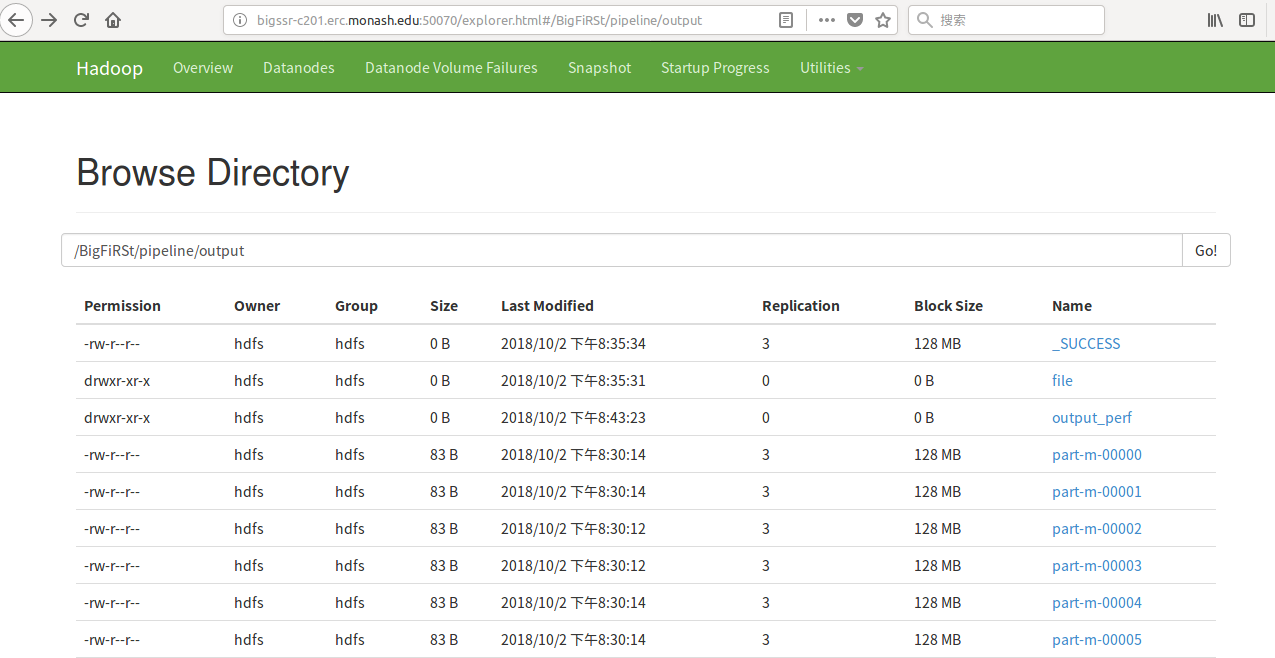


**Figure S12**. The output directory of BigFiRSt

Hadoop provide the specific command that can be used to download and view output files to personal computers. A glimpse of information in the index file is shown in **Figure S13**, where the first column represents instance ID of the map task, and the second column represents the path of the output of the map tasks for the reads merge operation. BigFiRSt does not use the reduce procedure by default, so the number of output index files is the same as the total number of maps. The details are as follows, where “/BigFiRSt/pipeline/output/output_perf/part-m-00000” is the path in HDFS and “./” is the local path.

| BigFiRSt$ Hadoop fs -get /BigFiRSt/pipeline/output/output_perf/part-m-00000 ./ |
| --- |


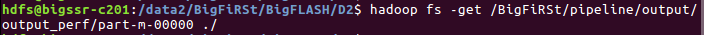


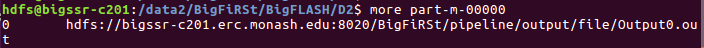


**Figure S13**. The index file of the BigFLASH module in BigFiRSt

The “file” folder contains result files of BigFLASH, show in **Figure S14.**


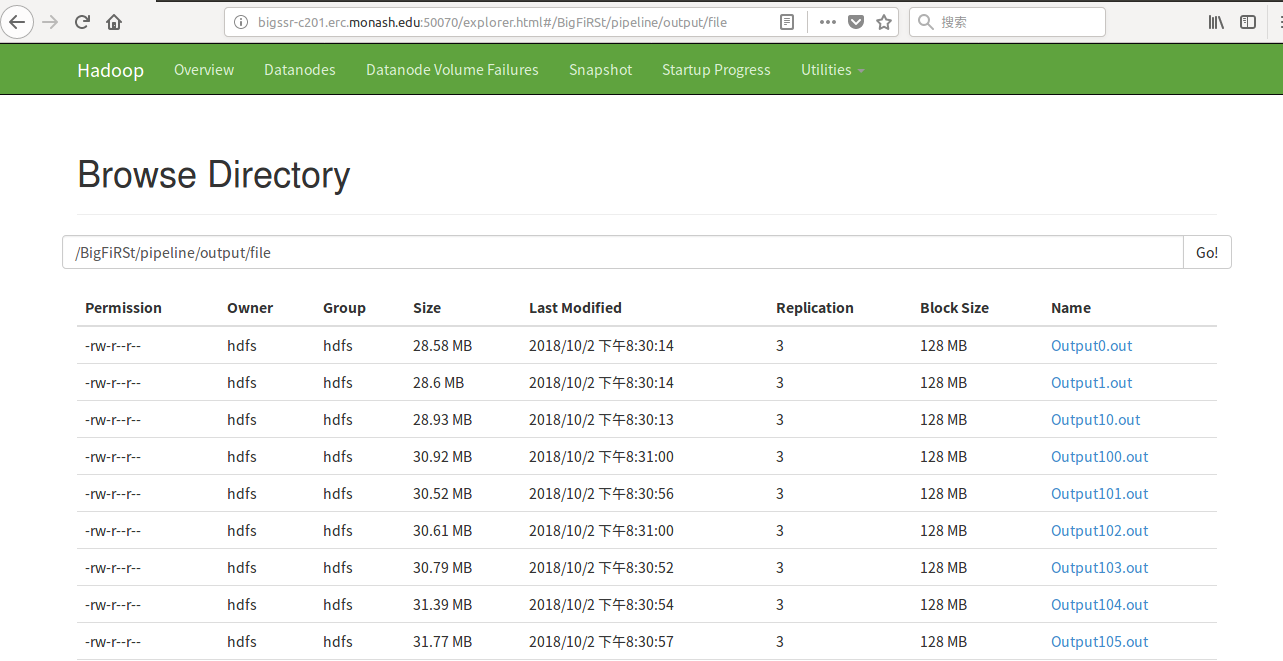


**Figure S14**. A list of the read merge results of the output of BigFLASH module when the reduce number is zero

A glimpse of the “Output0.out” file is shown in **Figure S15**.


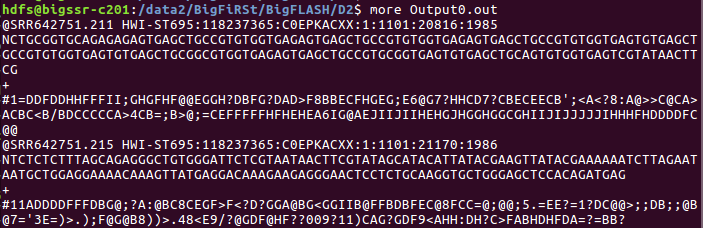


**Figure S15**. Results of the read merge of the BigFLASH module when the reduce number is zero

The “output_perf” folder contains the result files of BigPERF. This is shown in **Figure S16**.


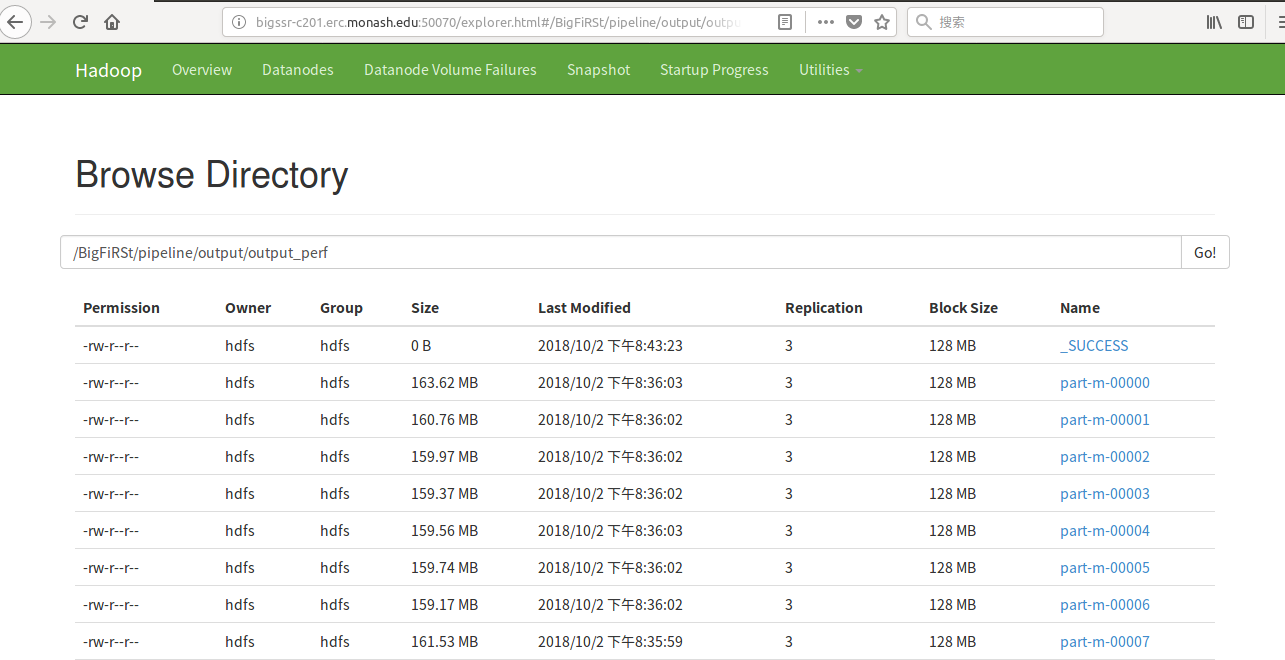


**Figure S16**. A list of the SSRs mining results of the output of BigPERF module when the reduce number is zero

The results of BigPERF module are generated in the path directory specified in the running parameter “-pho”. A glimpse of the information of the BigPERF module for SSRs mining results is shown in **Figure S17**, where: (a) the first column represents chromosome or sequence ID; (b) the second column represents the starting position of the mined SSR in the chromosome or sequence; (c) the third column represents the ending position of the mined SSR in the chromosome or sequence; (d) the fourth column represents the repeat Class grouped by their cyclical variations ; (e) the fifth column represents the total length of the mined SSR repeat sequence, (f) the sixth column represents repeat strand; (g) the seventh column represents the repeating time of the base motif, and (h) the eighth column represents the actual repeat. The details of the "part-m-00000" file are as follows:


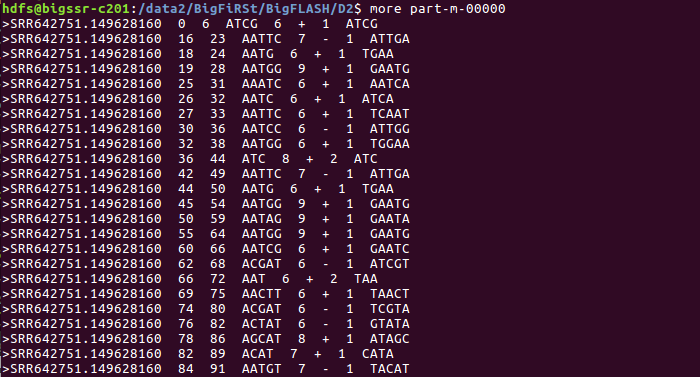


**Figure S17**. Results of the SSR mining of the BigPERF module

Of course, if the '-pa' parameter is specified when running BigFiRSt, an HTML analysis file will be generated [3]. An example of analysis result is shown in **Figure S18**.


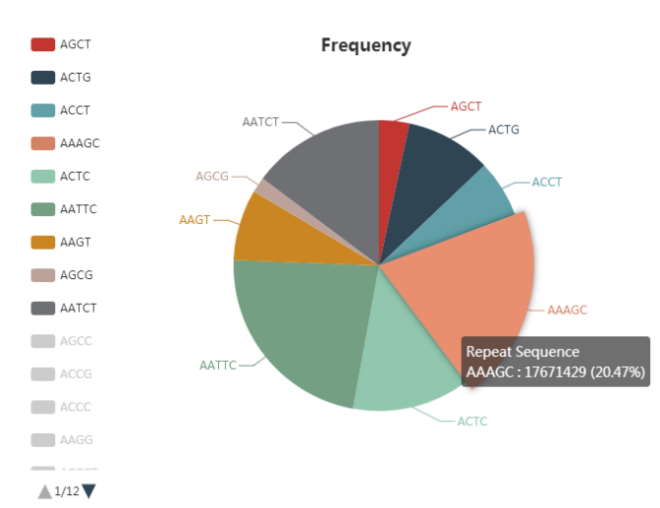

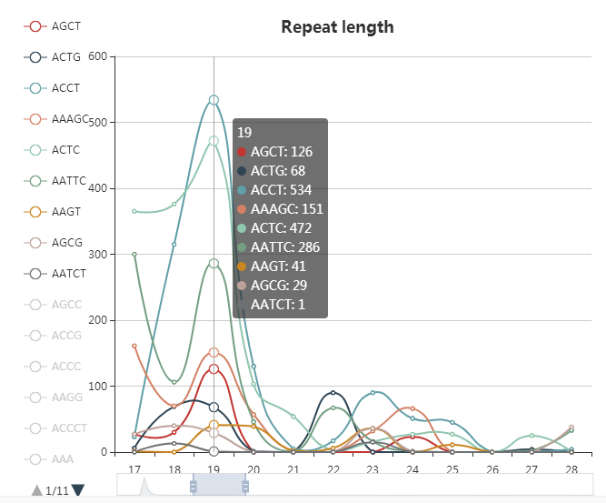


**Figure S18**. BigFiRSt performs SSRs mining results analysis report on D2` dataset. **(A)** Proportional distribution of repeats in D2` dataset. **(B)** Relationship between repetition length and repetition frequency in D2` dataset.

**1.2.3 BigFLASH usage**

Here we provide an example to illustrate how to run the BigFLASH. BigFLASH runs in five steps and the first four steps are the same as the BigFiRSt steps. Please refer to **Section 1.2.2** for details. Running command is as follows:

| BigFiRSt$ python BigFiRSt.py \  -bf \  -hh /data2/Hadoop \  -mH bigssr-c201.erc.monash.edu \  -cP 8020 \  -fj /data2/BigFiRSt/BigFLASH.jar \  -fmc com/nwafu/cjx/bigflash/BigFlashRunner \  -fi1 /data2/BigFiRSt/BigFLASH/D2/SRR642751_1.fastq \  -fi2 /data2/BigFiRSt/BigFLASH/D2/SRR642751_2.fastq \  -fhi hdfs://bigssr-c201.erc.monash.edu:8020/BigFiRSt/BigFLASH/D2/input \  -fho hdfs://bigssr-c201.erc.monash.edu:8020/BigFiRSt/BigFLASH/D2/output |
| --- |

The BigFLASH automatically conducts a series of operations such as data preprocessing, uploading, distributed computing, and merging read pairs. The user only needs to wait for the completion of the task. The details of the BigFLASH running in the Hadoop cluster are shown in **Figure S19**. The specific usage of the BigFLASH module parameters has been described in **Section 1.2.1**.


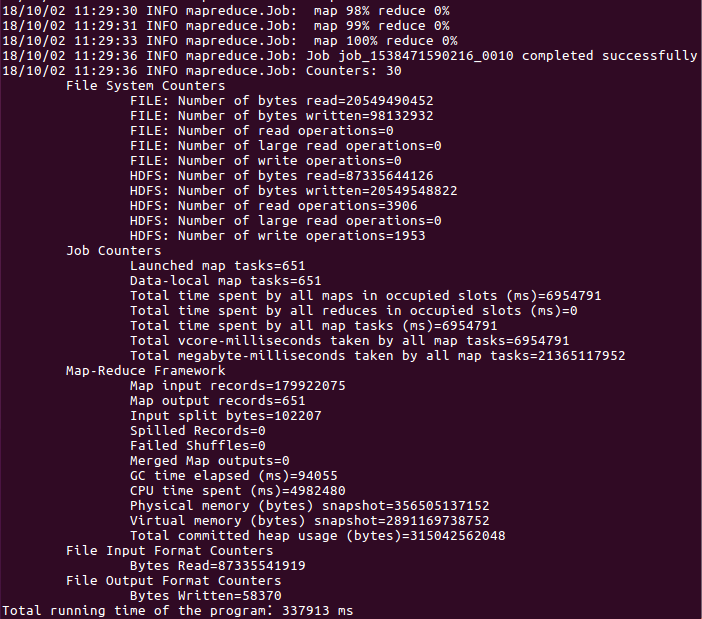

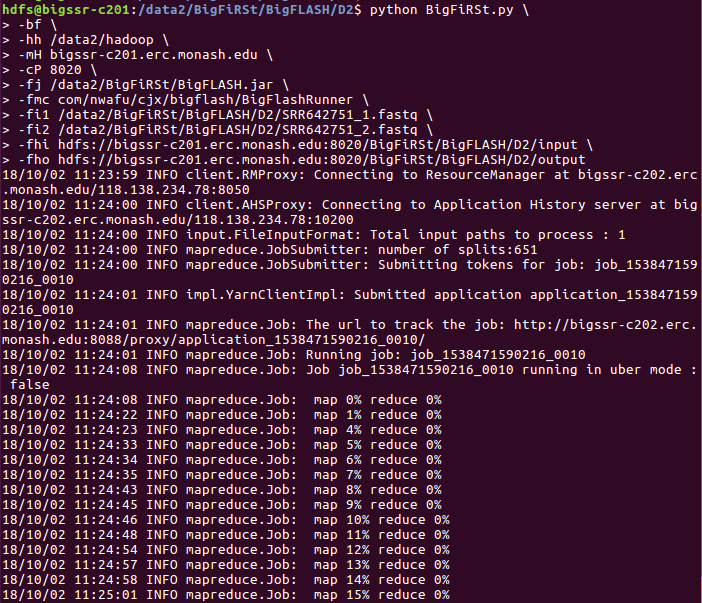


**Figure S19**. BigFLASH running in the Hadoop cluster

If “reduce” is set to zero, other web interfaces and output related information are similar to those described in **Section 1.2.2**. However, if “reduce” is not set to zero, the different interfaces will be as shown in **Figures S20 and S21**. In **Figure S20**, the “file” folder and the “part-r-0000*” file are the output of BigFLASH. The “file” folder is used to store the merge result files of the map phase, and the folder will be empty when the reduce phase is completed. The “part-r-0000*” file is a merged file of the output of BigFLASH.


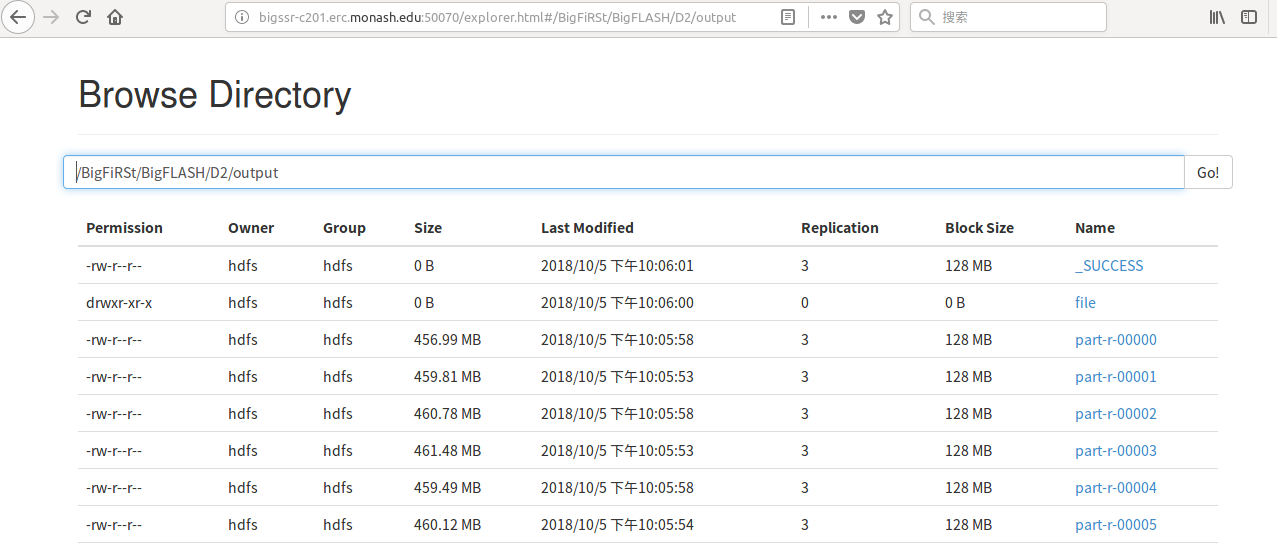


**Figure S20**. A list of the read merging results by BigFLASH when the reduce number is not zero


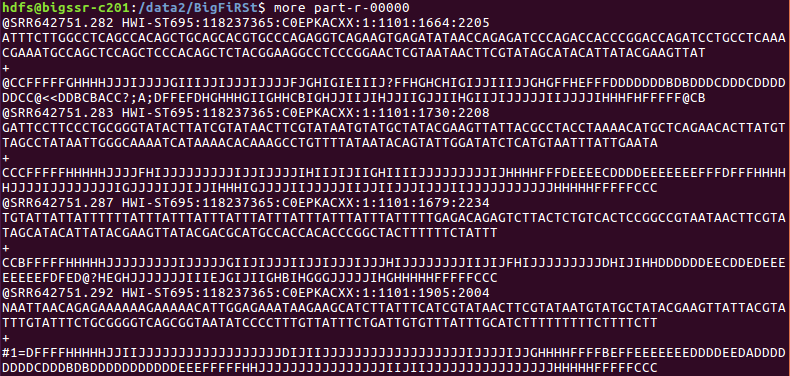


**Figure S21**. Contents of the read merging result by BigFLASH when the reduce number is not zero

**1.2.4 BigPERF usage**

Here we show an example to illustrate how to run BigPERF for mining SSRs. BigPERF runs in five steps and, the first four steps are the same as the BigFiRSt steps. Please refer to **Section 1.2.2** for details. Running command is as follows:

| BigFiRSt$ python BigFiRSt.py \  -bp \  -hh /data2/Hadoop \  -mH bigssr-c201.erc.monash.edu \  -cP 8020 \  -pj /data2/BigFiRSt/BigPERF.jar \  -pmc nwafu/cie/bigssr/BigPerfRunner \  -pi /data2/BigFiRSt/BigPERF/70779_ref_Adig_1.1_chrMT.fa \  -phi hdfs://bigssr-c201.erc.monash.edu:8020/BigFiRSt/BigPERF/input \  -pho hdfs://bigssr-c201.erc.monash.edu:8020/BigFiRSt/BigPERF/output \  -pm 3 -pM 5 -pl 6 -ps 0 -pS 2147483646 |
| --- |

BigPERF automatically conducts a series of operations such as such as data uploading, distributed computing and SSRs mining. The user only needs to wait for the completion of the task. The running details in the Hadoop cluster are shown in **Figure S22**. The specific usage of the BigPERF module parameters has been described in **Section 1.2.1**.

Other web interfaces and output related information are similar to those described previously. Please refer to **Section 1.2.2**.


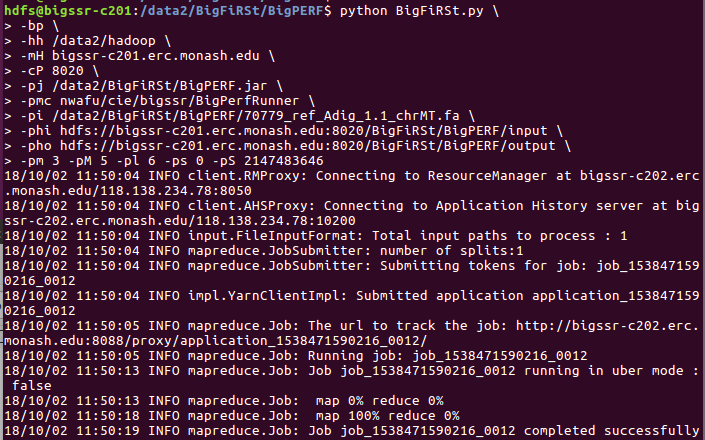


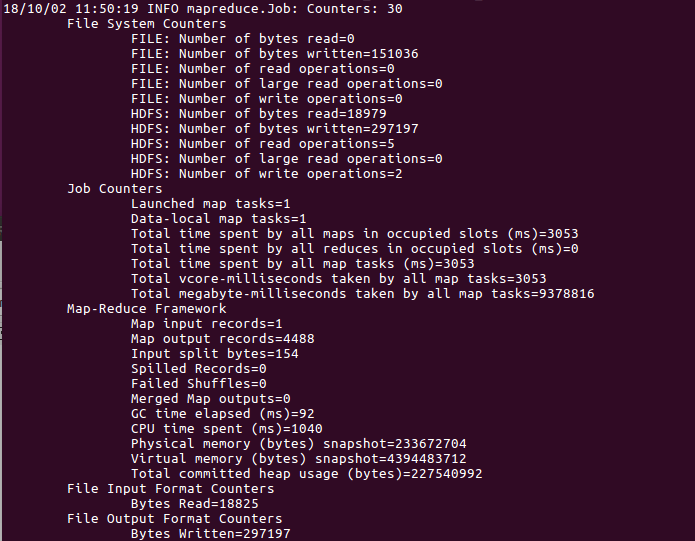


**Figure S22**. Operation information of the BigPERF module running in the Hadoop cluster

**1.3 Implementation details of shared library in BigFLASH**

Hadoop is implemented in Java language and provides many Java APIs for developing Hadoop applications. Generally, applications based on Hadoop are implemented in Java to achieve better interactive with Hadoop. However, the original FLASH algorithm is written in C programming. Like BigBWA [4], BigFLASH uses Java Native Interface (JNI) to integrate Java programming code with C programming code of FLASH. We note we only use FLASH source code to build a shared library file named with “libflash.so”. BigFLASH parses input parameters and passes them to the main method of FLASH by loading “libflash.so”. The workflow of building “libflash.so” is as follows (**Step 1-4**).

**Step 1:** Create a Java Class “FlashJni.java” that uses the C source codes of FLASH. In the Class “FlashJni.java”, a native instance method “FlashJni(int argc, String[] argv)” is declared. The method “FlashJni(int argc, String[] argv)” does not contain a body. This means that it is implemented in C language. The parameter “argv” in this method denotes the input parameters, and “argc” is the number of input parameters. The Class “FlashJni” and the native method “FlashJni(int argc, String[] argv)” are as follows.


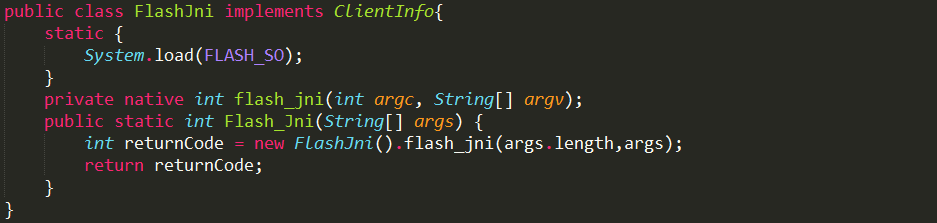


**Step 2:** Compile “FlashJni.java” and generate the C Header File “FlashJni.h”.

If users’ cluster installed JDK 8 or upper versions, users can use “javac -h” to compile “FlashJni.java” and generate C header file “FlashJni.h” as follows:

| BigFiRSt$ javac -h FlashJni.java |
| --- |


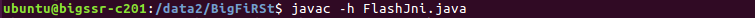


If users’ cluster uses older JDK versions (that is, lower than JDK 8), you can execute the following commands:

| BigFiRSt$ javac FlashJni.java |
| --- |


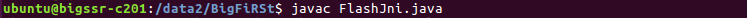


| BigFiRSt$ javah FlashJni |
| --- |


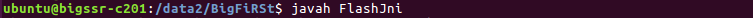


In “FlashJni.h”, a C function is declared as follows:


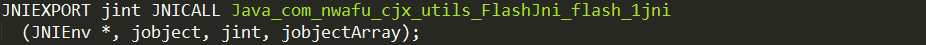


**Step 3:** Create the C program “FlashJni.c” which implements the declared method “Java_com_nwafu_cjx_utils_FlashJni_flash_1jni” in “FlashJni.h”.

In the method “Java_com_nwafu_cjx_utils_FlashJni_flash_1jni”, users only parse the received arguments (the input parameters required by FLASH) and pass them to the main method of FLASH.

**Step 4:** Compile “FlashJni.c” along with FLASH source code and generate a shared library file “libflash.so”.

1. **Web server**

In addition, we developed an online web server for BigFiRSt, to facilitate user to merge read pairs and/or mine SSRs in small scale datasets. This is freely available and accessible at <http://ptm.firstmetcs.com/BigFiRSt>. Two algorithms FLASH and PERF and the pipeline are provided in this web server too. To get result files, users can upload files and select desired parameters.

**2.1 Introduction**

Here, we show a short tutorial on how to use this web server.

The main page has six sections (Home, Service, Search, Job List, Download, Help, and Contact). The details are shown in **Figure S23**.


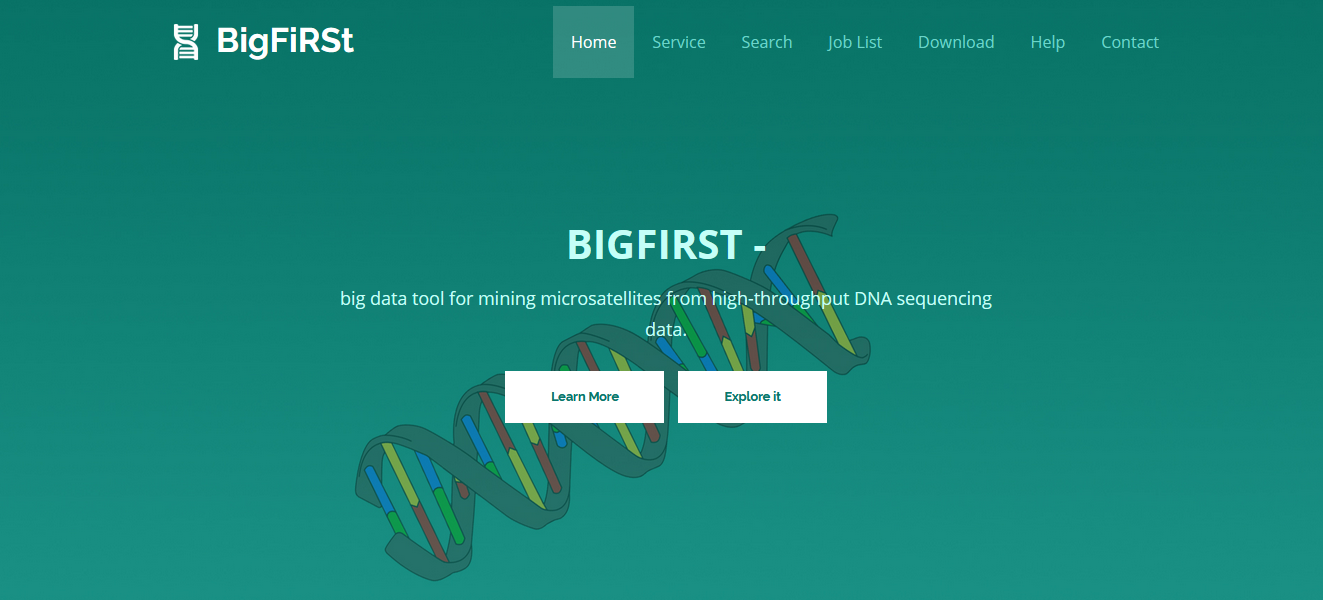


**Figure S23**. Main interface of the web server

1. Home: brief descriptions of the framework of BigFiRSt.
2. Service: provide the online operations of the FLASH, PERF, the pipeline.
3. Search: search the submitted job.
4. Job List: display all submitted jobs.
5. Download: download the source code of BigFiRSt and the datasets in our experiment.
6. Help: provides a guide to help user to use the web server.
7. Contact: methods for users to contact us if they have questions or would like to request more information about this website.

**2.2 Online FLASH usage**

Please refer to the following steps (Step 1 to 4) Online FLASH usage.

**Step 1:** Upload two FASTQ files or input sequences in the text area. For FLASH, users must provide a dataset by uploading two FASTQ files or input two FASTQ sequences. If you don’t know how to do this, you can just click “example”, and some sample sequence inputs will be showed, as in **Figure S24**. Moreover, users can also choose to upload two FASTQ files, and an illustration of this is shown in **Figure S25**. It's worth noting that you need to make sure the input box is empty before uploading the files. If it is not empty, please click ‘clear’ to clear the previously entered sequence and then upload the files.


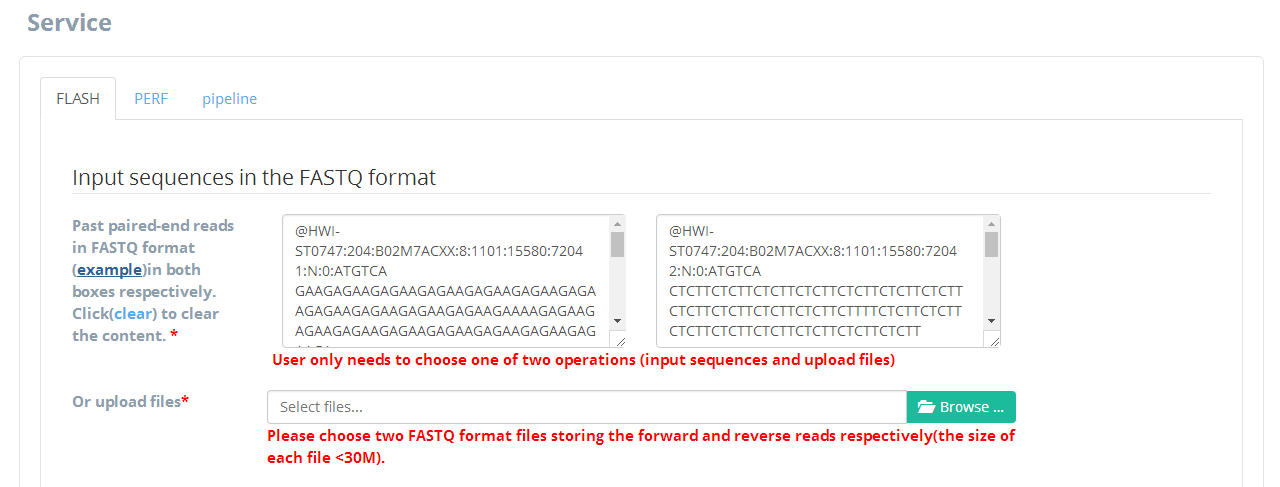


**Figure S24**. Input read pairs in FASTQ format


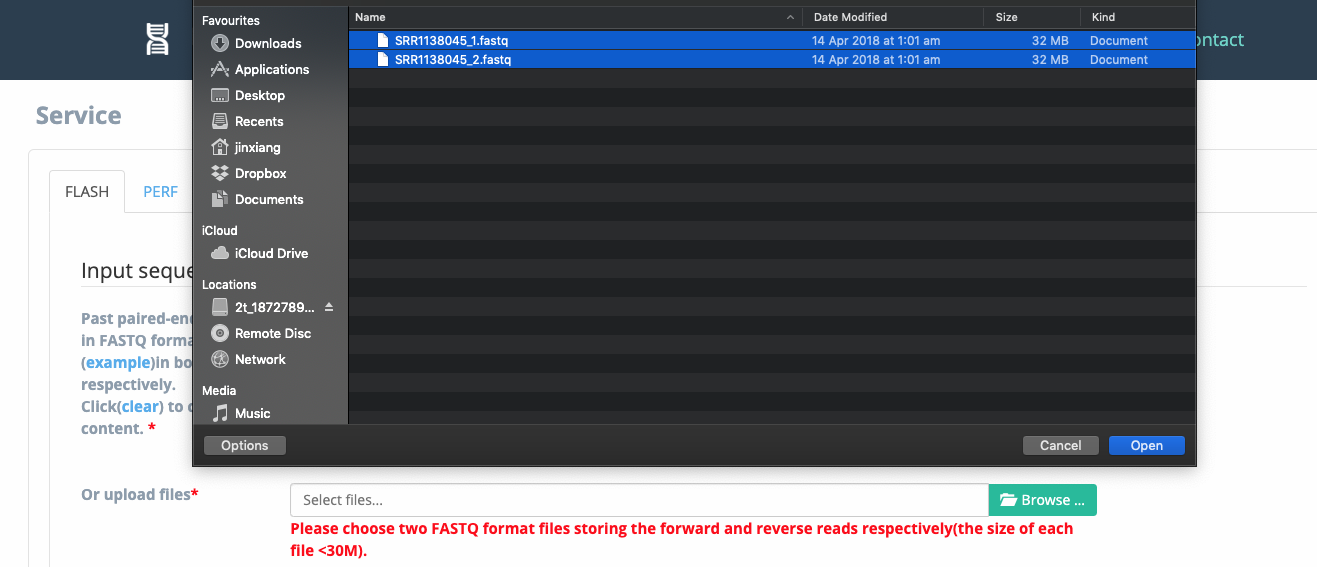


**Figure S25**. Uploading two files in FASTQ format

**Step 2:** Input values of different kinds of parameters in the designated text area and select descriptors. For convenience, there are many descriptor types to choose from. Text areas should be chosen or filled. For some options with checkboxes, if users want to apply these options, you can just check “YES”. For parameters with text area, users can just skip when they have no need to use them (that will be interpreted as a null value). The details are as shown in **Figure S26**.


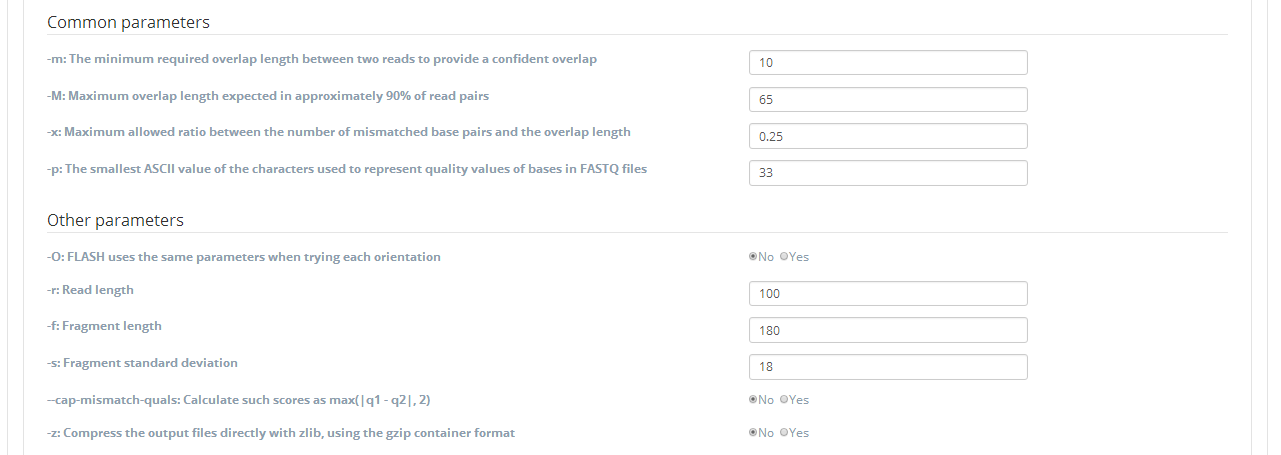


**Figure S26**. Input parameter values of FLASH algorithm

**Step 3:** Then you can click the “Submit” button at the bottom of the interface to run the FLASH algorithm. Alternatively, users can also provide their email addresses in order to receive a notification after the submitted job is finished. Finally, when the submitted job is completed successfully, users can view the job details and download the generated results. In this case, users will receive a notification email and can check the job details by clicking a hyperlink in the Email. The details are as shown in **Figure S27**.


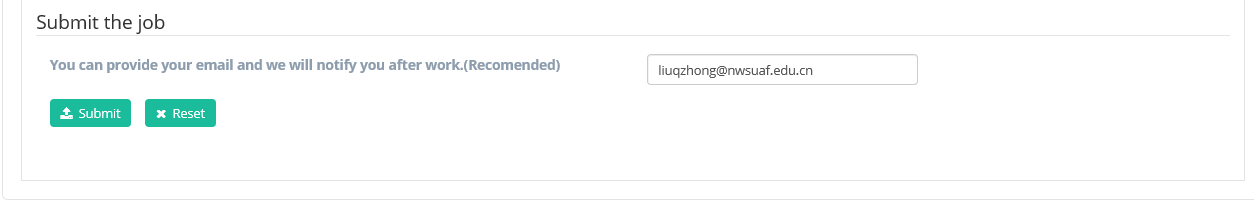


**Figure S27**. Submit job of FLASH algorithm

**Step 4:** Get the result files and download them.

After a few seconds, the interface will jump to the interface of download. The results with chosen descriptors are listed for download. The details are as shown in **Figure S28**.


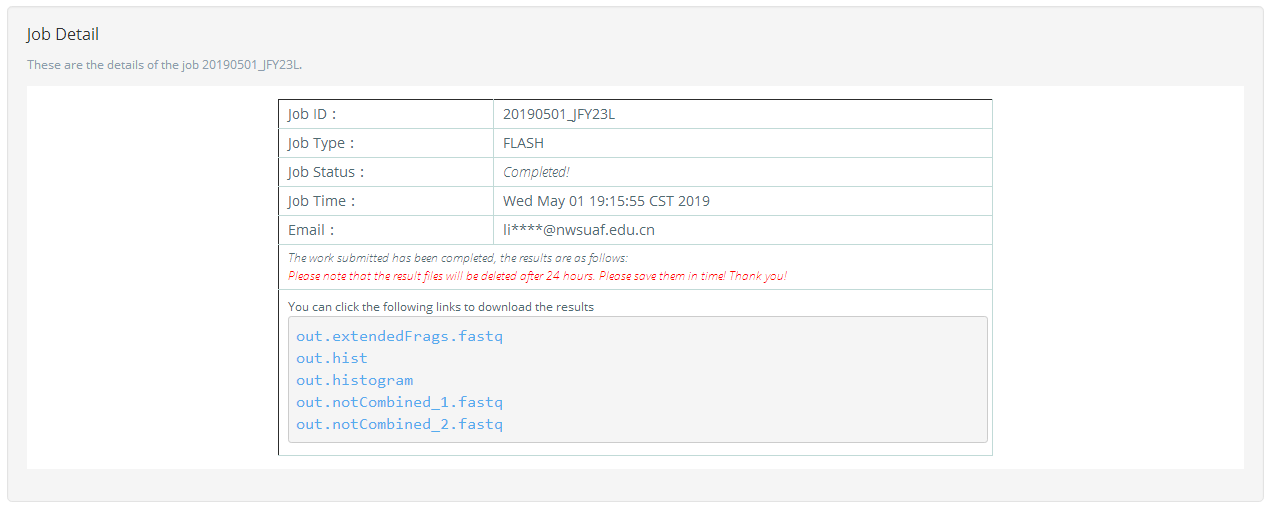


**Figure S28**. Download interface of FLASH algorithm

**2.3 Online PERF usage**

The first three steps are similar to the usage of online FLASH. An illustration is shown in **Figure S29-31**. After users submit the job, the statistical table of the result page is shown **Figure S32**. In the statistical table, users can input a preferred SSR, then the statistical table only remains the rows with the preferred SSR. In addition, users can download the complete result or the filtered result from this page.


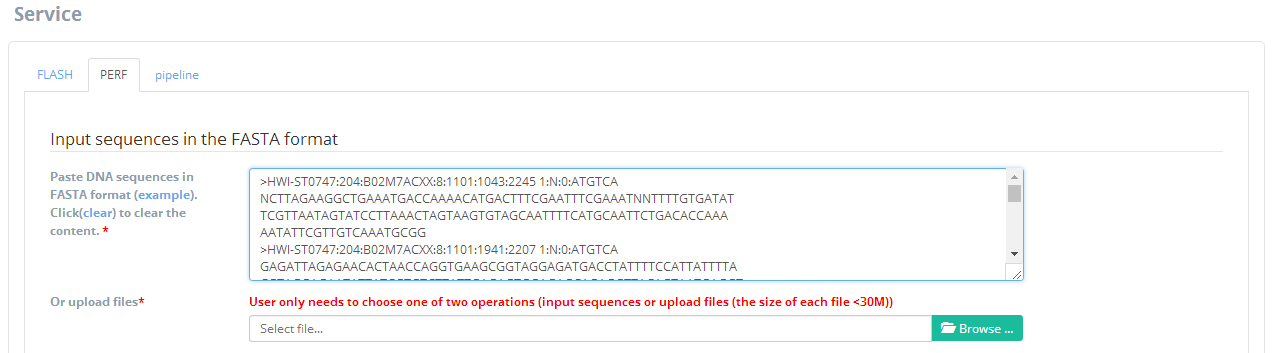


**Figure S29**. Input sequences with the FASTA format


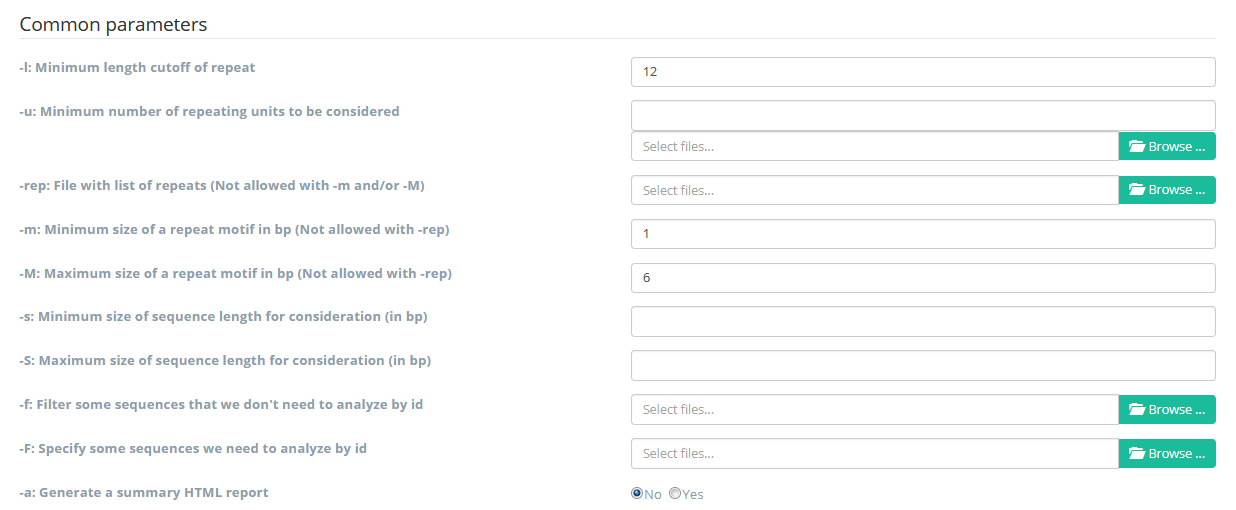


**Figure S30**. Input parameter values of the PERF algorithm


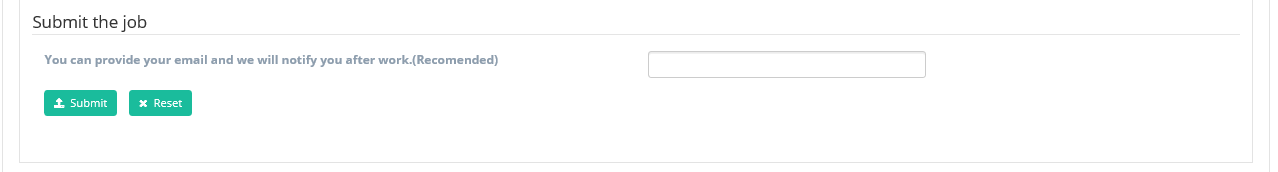


**Figure S31**. Submit job of the PERF algorithm


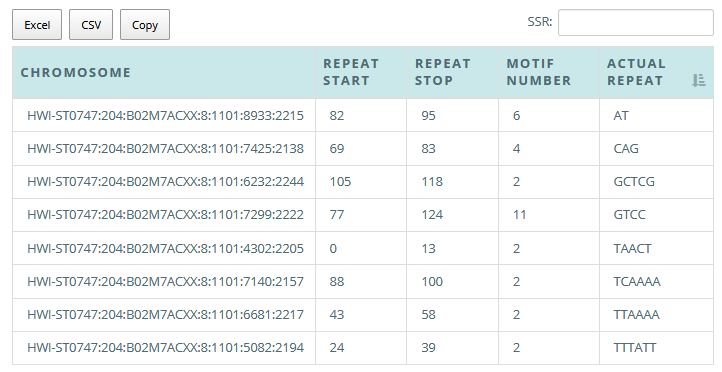


**Figure S32**. The statistical table of the result page of the PERF algorithm

**2.4 Pipeline**

This module integrates the FLASH algorithm with PERF into a pipeline. The process of the pipeline is similar to the usage of FLASH and PERF. Users first upload paired-end reads, then set the parameter values required by the FLASH and PERF, and finally submit this job. It needs to be emphasized that a new parameter about length of terminal flanking sequences is added in the pipeline.

References

1. Ghemawat S, Gobioff H, Leung ST. The Google file system. 2003.

2. Abecasis GAR, David A, Adam A et al. A map of human genome variation from population-scale sequencing, Nature 2010.

3. Li D, Mei H, Yi S et al. ECharts: A declarative framework for rapid construction of web-based visualization ☆, Visual Informatics 2018:S2468502X18300068-.

4. Abuín JM, Pichel JC, Pena TF et al. BigBWA: approaching the Burrows–Wheeler aligner to Big Data technologies, Bioinformatics 2015;31:4003.

1. <http://hadoop.apache.org/docs/stable/hadoop-project-dist/hadoop-common/ClusterSetup.html> [↑](#footnote-ref-1)
